# Food, Pregnancy & Me: Exploring food insecurity in pregnancy in the UK to inform future public health intervention needs–A mixed-methods study protocol

**DOI:** 10.1371/journal.pone.0321638

**Published:** 2025-05-07

**Authors:** Kiya L. Hurley, Kate Jolly, Heather Brown, Steph Scott, Zainab Akhter, Eleanor Dyer, Giang Nguyen, Amelia A. Lake, Christine Möller-Christensen, Nicola Flint, Angela Baker, Kerry Brennan-Tovey, Sonya Dickie, Emma Gibson, Catherine Jackson, Rachel Loopstra, Harbir Nagra, Judith Rankin, Dianne Williams, Alice Wiseman, Nicola Heslehurst

**Affiliations:** 1 Department of Applied Health Sciences, University of Birmingham, Birmingham, West Midlands, United Kingdom; 2 Public Health Research for Health (PHRESH) Consortium, West Midlands, United Kingdom; 3 National Institute for Health and Care Research (NIHR) Applied Research Collaboration West Midlands, West Midlands, United Kingdom; 4 Division of Health Research, Lancaster University, Lancaster, Lancashire, United Kingdom; 5 LiLaC Consortium, United Kingdom; 6 Population Health Sciences Institute, Newcastle University, Newcastle upon Tyne, Tyne and Wear, United Kingdom; 7 Fuse, The Centre for Translational Research in Public Health, Newcastle upon Tyne, United Kingdom; 8 School of Health and Life Sciences, Teesside University, Middlesborough, North Yorkshire, United Kingdom; 9 Research and Development/ Maternity Department, Gateshead Health NHS Foundation Trust, Gateshead, Tyne and Wear, United Kingdom; 10 University Hospitals Coventry and Warwickshire NHS Trust, Coventry, West Midlands, United Kingdom; 11 Coventry City Council, Coventry, West Midlands, United Kingdom; 12 NIHR Applied Research Collaboration North East and North Cumbria, United Kingdom; 13 Felling Food Network, Gateshead, Tyne and Wear, United Kingdom; 14 Gateshead Council, Gateshead, Tyne and Wear, United Kingdom; 15 Department of Public Health, Policy and Systems, University of Liverpool, Liverpool, Merseyside, United Kingdom; 16 Moat House Community Trust and Coventry Feeding Network, Coventry, West Midlands, United Kingdom; PLOS: Public Library of Science, UNITED KINGDOM OF GREAT BRITAIN AND NORTHERN IRELAND

## Abstract

**Introduction:**

There are several known risks relating to poor nutrition during pregnancy, including the development of complications and poor birth outcomes. While food insecurity is associated with poorer nutrition, data on the prevalence and severity of food insecurity in pregnancy in the UK is lacking. This study aims to explore the prevalence, experiences and health impact of food insecurity in pregnancy in England to develop strategic recommendations for intervention strategies.

**Methods and analysis:**

Food, Pregnancy & Me is an observational, multi-method study. Questionnaires exploring diet quality, food security, mental health, and other health behaviours will be distributed to all women and pregnant people in their third trimester in two NHS Trusts in England (North East and West Midlands). Returned questionnaires (n=605) will be linked to routine maternal and birth outcome data and pseudo-anonymised. We will estimate the prevalence of food insecurity in pregnancy in these locations, associations with diet quality, maternal mental health, and pregnancy outcomes (e.g., pre-term birth, pre-eclampsia, gestational diabetes). Qualitative interviews (n=40) with participants identified as having experienced food insecurity will explore their lived experience, support received, and recommendations for additional support needs. Through a series of co-production workshops with local and national system shapers and experts by lived experience, we will use the data gathered to produce strategic recommendations for intervention with pregnant women and people facing food insecurity. We will then explore the potential costs and benefits of implementing the proposed recommendations.

**Ethics and dissemination:**

Ethical approval was obtained from Newcastle and North Tyneside 1 NHS Research Ethics Committee (24/NE/0027). Findings will be disseminated to key national and local system shapers and policy makers, advocacy groups, and the public through reports, presentations, the media and open access publications.

**Study registration number:**

ISRCTN16655955

## Introduction

Food insecurity (FI) exists when a person lacks regular, socially and culturally acceptable, access to safe and nutritious food for normal growth and development and an active and healthy life [1]. FI prevalence has increased across high-income countries, and the United Kingdom (UK) is no exception [2]. Monitoring households in the UK estimates the prevalence of moderate/severe FI to be around 10%, with a further 7% experiencing marginal food security [3], with substantial geographic and socio-demographic variations. For example, higher rates are seen in households with children (especially those with more than three children), amongst those households with a person limited by a disability, in minoritised ethnic groups, and living in the North East of England [4,5].

FI is associated with nutritionally inadequate diets, which are often high in energy-dense and high fat, salt and sugar, processed food [6–8]. The health impact of a poor diet on an individual has been well established, with increased risk of developing various chronic diseases, including cardiovascular disease and Type 2 diabetes [6–13]. FI has been further associated with high levels of stress, anxiety, depression and sleep difficulties [14–20].

Pregnancy is a life course stage with increased nutritional requirements to support the health of the mother and the development of the infant [21,22]. This means that pregnant women or people, and their children, are particularly vulnerable to the physical and mental health impacts of FI. The consequences of suboptimal nutrition during pregnancy can be significant, in the short- and long-term, for both mother and child. FI during pregnancy has been shown to be associated with a range of adverse outcomes such as gestational diabetes mellitus, mood disorders, inadequate or excessive weight gain, and neonatal mortality [23,24]. In addition, research has highlighted that epigenetic modifications which occur due to inadequate nutrition in pregnancy can have life-long implications for the infant, including an increased risk of obesity, cardiovascular disease and Type 2 diabetes. [25–29]. Despite these stark health implications, research with women in households experiencing FI reports coping mechanisms such as women restricting their own food intake in favour of their children and other household members [19,20,30–32]. Public health policy and interventions are needed during this key life course stage to ensure availability of nutritious culturally appropriate food, to promote the best start in life, and to mitigate the harmful impacts of environmental exposures to inequalities which occur in utero, such as FI.

Whilst FI in England is currently monitored at the population level by various Government departments [3,33] and charities [5,34], little is known about the current prevalence, drivers and mitigators of FI during pregnancy in England. Questionnaires used for FI monitoring through nationally representative surveys do not include questions about respondents’ pregnancy status, and even if such questions were included, numbers would likely be too small to obtain reliable prevalence estimates. Potential pregnancy-related factors are also not captured. As a result, most of the evidence on food insecurity and pregnancy to date comes from the USA and Canada [23,35]. This study aims to determine the prevalence of FI in two geographically and socio-economically diverse regions of England, to understand the experiences and health impacts of those who have faced FI during pregnancy and establish policy and practice recommendations to address FI during this crucial life course stage.

## Methods

### Study aim and objectives

This study aims to explore the prevalence, experiences and health impact of food insecurity in pregnancy in England and co-develop strategic recommendations for intervention strategies. This will be achieved by:

1. Quantitatively measuring FI in the third trimester of pregnancy to estimate prevalence and its association with socio-demographic characteristics (Phase I).
2. Linking pregnancy FI data with routine pregnancy health records for the mother and the baby to estimate associations between FI (and severity) with maternal and child health (Phase I)
3. Quantitatively collecting maternal diet quality data in the third trimester to explore associations between FI (including severity) and maternal diet quality (Phase I)
4. Qualitatively exploring the experiences of women experiencing FI in pregnancy and their support/intervention needs (Phase II)
5. Working with a range of stakeholders/system shapers, and the evidence generated in phases I and II, to co-produce strategic recommendations for holistic provision of support for women and pregnant people experiencing FI during and after pregnancy (Phase III).
6. Assessing the costs and benefits of implementation of the strategic recommendations for intervention support to help decision makers plan future services and interventions (Phase III)

### Study design and setting

**Fig 1:**
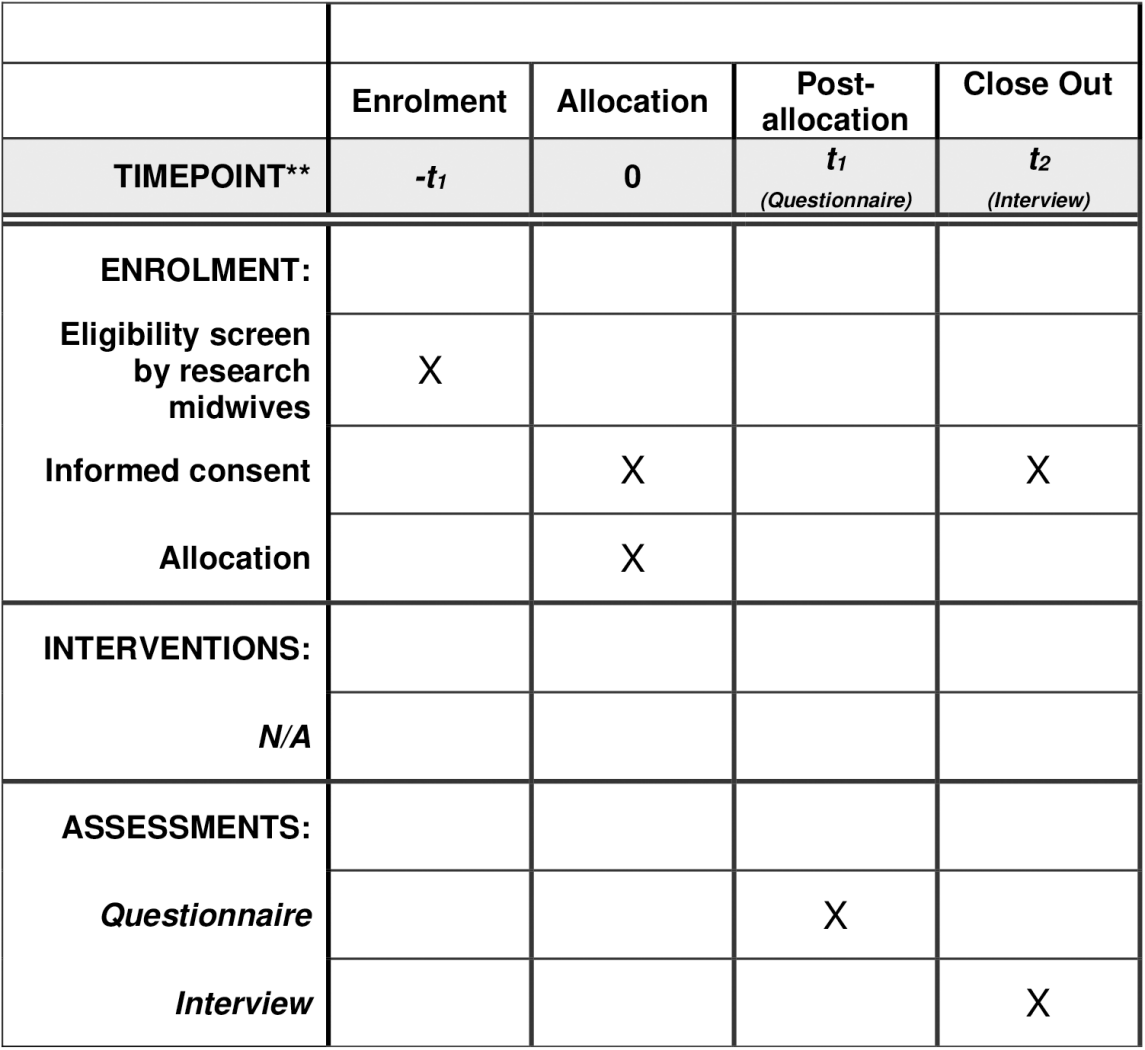
SPIRIT schedule of enrolment, interventions, and assessments.

This observational, multi-method study, consists of three phases. Phase I is a survey of women and pregnant people in their third trimester. Participants in their third trimester will be able to report experiences of food insecurity throughout their pregnancy. Data will be collected on their diet quality, food security, mental health, and tobacco/e-cigarette use. Survey data will be linked to data from routine maternity records, including data on maternal health during pregnancy (e.g., pre-eclampsia, gestational diabetes) and childbirth outcomes (e.g., birth gestation, birth weight, and Apgar score). Phase II involves interviews with those identified by the research team from the survey response as having experienced FI. The interviews will explore their lived experience, the support they received, and their recommendations for additional support needs when facing FI in pregnancy. Fig 1 shows the SPIRIT schedule of enrolment, interventions, and assessments for participants in Phases I and II.

**Fig 2:**
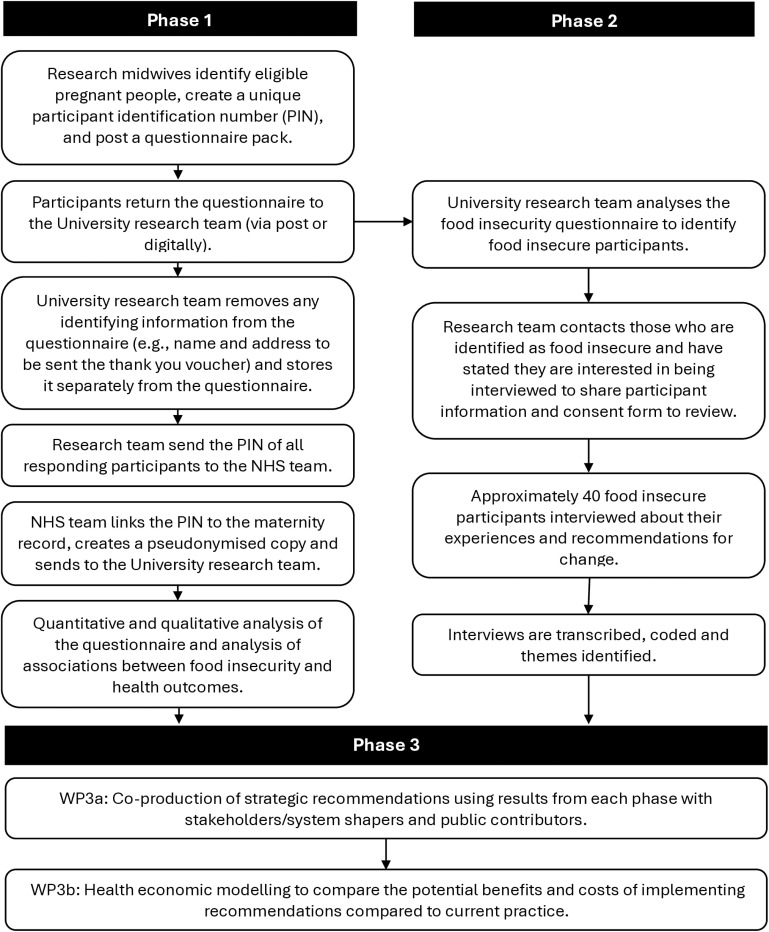
Flow of study phases.

Phase III will co-produce strategic recommendations for interventions and services working with women and pregnant people facing FI and determine the costs and benefits to implementation of these recommendations compared to standard practice. Fig 2 shows the flow of the study phases.

### Study setting

The study will be conducted in two NHS Trusts: one in the North East and one in the West Midlands of England. These regions were chosen due to their high prevalence of FI, deprivation, and demographic diversity. For example, compared to other regions in England, the North East has the highest prevalence of FI (12% experiencing low/very low food security, compared to 6% in the East of England) [36] and the West Midlands has over 40% the population from ethnicities other than White British (compared to a national average of 18%) [37,38].

### Phase I: The survey

This will be a prospective study with data linkage to routine maternity records. The survey will include quantitative data that are not routinely collected by maternity services and will be linked to maternal and child health record data with a unique ID number for each participant. Data linkage to routine maternal and child health records will take place following delivery and discharge from maternity care. All routine maternity data will be pseudo-anonymised using the unique participant IDs before sharing with research team members outside of NHS Trusts.

### Eligibility criteria

All adult women and pregnant people in their third trimester of a viable pregnancy (28–40 weeks gestation) who register for maternity services based at Queen Elizabeth Hospital Gateshead or University Hospital Coventry will be invited to take part in the study. Those under 16 years old, non-residents in the UK, less than 28 weeks gestation, post-partum, or receiving care by maternity services other than those based at Queen Elizabeth Hospital Gateshead or University Hospital Coventry, will be excluded from the study. Survey respondents will receive a £20 voucher upon completion of the questionnaire.

### Sample size

As prevalence of FI in pregnancy has not previously been explored in the UK context, accurate prevalence data on which to base sample size calculations is not available. We have therefore used the following prevalence estimates to inform the sample size calculations: FI prevalence of up to 30% (extrapolated from UK general population estimates which use different measurement tool for FI than in our study [5]); birth per year of 1800 at Gateshead Health Foundation Trust and 6000 per year at University Hospitals Coventry and Warwickshire [39]; 10% prevalence of antenatal depression [40]; and 7–8% prevalence of pre-term delivery [41]. Based on these estimates, and a confidence level of 95%, margin of error of 5%, and estimated attrition rate of 4%, the required sample size is 605 participants. We will aim to achieve approximately equal numbers of participants at each site.

### Recruitment

Each NHS Trust will distribute recruitment packs to all women and pregnant people entering their third trimester in their locality. The recruitment pack will contain a cover letter and information on how to obtain the study information in other languages, participant information sheet (PIS), questionnaire, pen, self-addressed envelope, and a leaflet highlighting local food aid, financial support and mental health support services. There will also be a QR code and website link to a participant information video to support those with low English literacy levels. The questionnaire will also be available to complete online. The rationale for this sampling technique is to be inclusive of all adult pregnant people and obtain information on the prevalence of FI in this population. To enhance recruitment, the study will also be promoted in maternity unit waiting areas via posters and targeted reminders sent through NHS communication routes (e.g., text messages or usual maternity notes application push notifications).

The challenges of recruiting participants reflective of the background pregnant populations in these locations has not been underestimated. Aggregated demographic statistics on the maternity population for the previous year will be extracted by the maternity teams in each NHS Trust. The demographic characteristics of survey respondents will be regularly monitored and compared against these aggregated statistics. Where imbalances arise, these will be discussed with the study management group (SMG) and study steering committee (SSC) to determine if supplementary recruitment methods should be employed. These supplementary methods include:

- Face-to-face recruitment for those attending general hospital-based appointments.
- Face-to-face recruitment from community midwife clinics located in areas where under-represented groups receive their care (e.g., areas of highest deprivation or geographies with high case load of minoritised ethnic groups).

### Outcome data collection

Return of the questionnaire will act as consent for inclusion in both the survey analysis and data linkage to maternity records and is made explicit in the participant information sheet and the questionnaire cover page.

The primary outcomes for this study are maternal antenatal depression and infant pre-term delivery (<37 weeks gestation). As antenatal depression assessments are only routinely implemented in standard care where depression is indicated on the Whooley screening questions, the Edinburgh Postnatal Depression Scale [42] will be included in the questionnaire to ensure an assessment is available for all participants. Infant pre-term delivery (less than 37 weeks gestation) will be obtained from routine maternity records.

To obtain an estimate of the prevalence of FI in this population, participants will complete the 10-item USDA Food Security Survey Module (USDA FSSM) using a 30-day recall period [43]. This questionnaire was developed in the USA and has been validated and used in different countries/contexts, including the UK [44]. Additional questions on FI stability, barriers to utilisation of food, and food coping strategies specific to pregnancy will explore further aspects of the FI experience among women and pregnant people [45].

Further data will be collected using the following questionnaires:

- *Diet quality:* An adapted version of the Brief Diet Quality Assessment Tool (BDQAT) [46] will be used. Adaptations to the original tool were made, in consultation with experts by lived experience to ensure acceptability to a variety of people, including those who are on a low-income, from a minoritised community, or pregnant. Adaptations included, adding sausages and burgers (animal source unspecified), removing alcohol, and adding example food items that are more commonly consumed in several minoritised communities in the UK (e.g., different types of bread (lavash, kulcha, chapatis) and sweet foods (jalebi, halwa, sweet valenki)). Open ended questions have been included to enable participants to describe any differences in their diet during pregnancy and in the preconception period.
- *Socio-demographic characteristics:* Routine maternity data does not include many of the individual level socio-demographics that would be relevant to the food insecure context. Questions relating to education, employment, receipt of benefits (including those specific to pregnancy such as healthy start vouchers, and more general benefits such as universal credit, personal independence payment), income, support networks and household occupancy will be included.
- *Health behaviours:* Questions related to health behaviours relevant to pregnancy have also been included. These include questions about smoking, vaping, and intentions regarding breastfeeding.

### Data linkage

Routinely collected relevant data will be exported from the electronic patient records and linked using NHS numbers and unique participant identification numbers for each participant. Table 1 shows the data that will be extracted from routine records.

**Table 1:** Data extracted from maternity records to be linked to the survey data.

Maternal Outcomes	Child outcomes
Gestational diabetes	Birth weight
Preeclampsia	Gestational age at delivery
Pregnancy induced hypertension	Large- and small-for gestational age
Induction	Breastfeeding initiated/at discharge from maternity services
Mode of delivery	Admission to special care baby units (and length of stay)
Length of stay in maternity unit	Apgar score at 1 and 5 minutes
Age	
Parity	
Body Mass Index	
Ethnic group	
Deprivation score (index of multiple deprivation (IMD) scores)	
Alcohol and illicit drugs	
Any relevant previous medical history (e.g., history of depression, previous pregnancy health such as low birth weight)	

### Data analysis

A binary food security status will be calculated for each participant according to the thresholds described by the USDA Household Food Security Survey Module [43]. Additionally, an ordinal variable will be calculated to determine sub-groups of high, moderate, low, and very low food security. Diet quality scores will also be calculated. The Edinburgh Postnatal Depression Scale will also be scored and binary variable will be generated with scores above 12 indicating antenatal depression.

Multivariable logistic regression models (binary and ordinal) will be used to examine associations between food security status and severity with health outcomes in pregnancy. The models will include socio-demographic and clinical factors related to both food security and health outcomes in pregnancy, such as age, deprivation score, ethnicity, employment status, smoking status, and household occupancy. We will explore differences between food security and maternal and child outcomes across socio-demographic characteristics by adding the relevant interaction terms to the developed models. If more than 5% of data are missing from demographic variables, multiple imputation methods will be used and sensitivity analyses conducted. All analyses will be carried out in STATA v.18.

Furthermore, open-ended questions included in the questionnaire will be analysed thematically to explore the impact of food security status in relation to pregnancy diets and health outcomes.

### Phase II: Qualitative interviews

Qualitative semi-structured interviews will be conducted with people in the perinatal period identified as having experienced FI through the response provided in the questionnaire. Interviews will explore participants’ lived experiences of FI during pregnancy, and what support they would have found most useful during and after pregnancy. Interviewees will be actively encouraged to consider support measures that could be implemented locally beyond/in addition to monetary support, and any potential spillover effect to the wider household. Participants in Phase II will receive a further £25 voucher.

All interviews will use a topic guide developed iteratively across the study. The topic guide was initially informed by the literature and discussions with public advisors with direct experience of FI in pregnancy. We will also review the survey data for topics that warrant further qualitative exploration.

### Eligibility, sample size, and recruitment

Within the questionnaire participants will be asked if they would be happy to be contacted to take part in the qualitative study (Phase II). Respondents who are identified as food insecure and have provided contact details for the purpose of discussing their experiences during their pregnancy will be identified. Purposive sampling will then be used according to respondents’ geographical region, socio-economic status, level of FI, and ethnic group. As participants will be asked to indicate their income and benefit status within the survey, it is anticipated that socio-economic status will be measured according to these indicators. Where this information is not available, IMD score will be used as a proxy.

Following recommendations for pragmatic assumptions around sample size [47], we anticipate that approximately 40 people will be interviewed (20 per geographical site). Sample size will be guided by the breadth and focus of the research questions; the demands placed on participants; the depth of data likely to be generated; pragmatic constraints; and the analytic goals and purpose of the overall project. Interviews will take place in-person or remotely, according to the needs of the project and participants. It is anticipated that all interviews will last approximately one hour. Consent will be given either in written format (in person interviews) or via an audio-recording (remote interviews). All consent files will be stored separately to the interview data.

### Data analysis

Interviews will be audio-recorded, transcribed and anonymised. It is anticipated that data will be analysed using software such as Nvivo 14 (QSR International, Melbourne, Australia) or similar, using reflexive thematic analysis [48]. Descriptive themes will be compared to identify patterns, similarities and differences in the data, and relationships between them elaborated, to generate analytical themes, and a consistent interpretation of the whole dataset. Themes will be discussed and challenged at regular project meetings, using a process defined as pragmatic double coding [49].

### Phase III: Co-production of strategic recommendations and economic analysis

Phase III has two parts. First, co-production methods will be used to generate the strategic recommendations arising from the data in Phases I and II. Discussion and refinement of themes emerging from the data with experts by lived experience and key stakeholders/system shapers (e.g., those working in practice, policy and voluntary, community, faith, and social enterprises to support those experiencing FI during pregnancy) will be conducted for each work package separately. Convergence coding matrix [50,51] will then be used to triangulate the results, identify areas of agreement and disagreement, and develop strategic recommendations for support for those experiencing FI during pregnancy. This analysis and resultant recommendations will then be discussed and prioritised with experts by lived experience and key stakeholders/system shapers (Phase IIIa). We will employ pre-trial health economic modelling to estimate the likelihood of cost-effectiveness to support decisions regarding a future clinical trial or implementation and identify potential improvements from the recommendations (Phase IIIb) [52]. Speaking with experts at the workshops at the start of Phase III, we will identify hypothesised outcomes (e.g., improved nutritional status and how this would translate into QALYs gained), for which types of women (e.g., by demographic characteristics) and the potential costs of delivering the proposed recommendations. This information will be used to estimate an Incremental Cost Effectiveness Ratio (ICER) comparing the QALYs gained for the recommendations compared to current practice vs the costs to determine if any of the recommendations are likely to meet cost-effectiveness thresholds (in the UK £20,000 per QALY) to ultimately be implemented. Next, we will use an Expected Value of Sample Information Approach to estimate the expected cost-effectiveness of conducting a trial of the proposed recommendations before widespread implementation [53–56].

### Ethical considerations

Full ethical approval for the study was obtained from the North East – Newcastle and Northern Tyneside 1 NHS Research Ethics Committee (REC) on 04^th^ April 2024. Any substantial amendments will be submitted to the REC and Health Research Authority (HRA) for approval before implementation. The REC and HRA will also be notified of any non-substantial amendments to the protocol.

### Data management and oversight

Newcastle University is the Study Sponsor and data controller and assumes overall responsibility for the study. Data management and storage is compliant with the UK Data Protection Act 2018 and follows the relevant Newcastle University policy and procedures. Following the publication of the main study results, anonymised data will be stored securely for a minimum of 10 years.

An independently chaired SSC has been convened to oversee the study. Membership comprises five independent academics with relevant expertise, an obstetrician, public representatives, and the Chief Investigator.

### Status and timeline of the study

Quantitative (Phase I) and qualitative (Phase II) data collection commenced at both sites by 15^th^ July 2024 and will complete on 30^th^ April 2025. Phase III will commence on 30^th^ April 2025 and complete on 30^th^ September 2025.

## Discussion

### Limitations of the study design

Despite the use of rigorous mixed qualitative and quantitative methods and a commitment to equality, inclusion and diversity, we anticipate some challenges. Consultation with our public advisors led to several modifications to our survey to reduce the barriers to completion, for example providing a pen alongside the paper survey and offering supported completion for those with low English literacy levels or physical limitations that may limit their ability to independently complete the survey in Phase I. The study team have included a cover sheet, translated into the eight most common languages in the localities, with instructions on how to access additional support for questionnaire completion. Dietary quality assessment in this population is difficult for several reasons including the ethnic diversity of the population being sampled, the reduction in alcohol intake during pregnancy (often included in dietary quality indices), and the need for brevity stressed by our public advisors. As such, a short dietary quality index, developed based on UK consumption data [46], has been adapted for this population to include a greater variety of example foods from other cultures. However, this tool will provide limited nutritional information.

Recruitment for Phase II in the first instance is through the completion of the Phase I survey and provision of consent to be contacted. The team acknowledge this may be challenging. However, in the event of poor recruitment of eligible participants, ethical approval has been granted to diversify and target recruitment methods, e.g., using community researchers.

### Dissemination

Study findings will be shared with academic, practice and policy audiences through a variety of mechanisms, including open access publications and research and policy briefings. Further plans for wider dissemination will be refined in conjunction with the study’s public advisors to determine the most suitable mode and appropriate messaging of the study findings for a wider audience. A full report of the study will be published in the NIHR Journals Library.

## Supporting information

S1 File SPIRIT-OutcomesChecklist - Food, Pregnancy & Me. (PDF)

S2 File FPM_Protocol_V3.0_16-April-2024. (DOCX)
