# Supplementary material for: Food, Pregnancy & Me: Exploring food insecurity in pregnancy in the UK to inform future public health intervention needs–A mixed-methods study protocol
Source: PLoS One. 2025 May 7;20(5):e0321638. doi: 10.1371/journal.pone.0321638 (PMC12058193; doi:10.1371/journal.pone.0321638)
Supplement: S2 File — (DOCX) [file pone.0321638.s002.docx]

Study Protocol

**Food, Pregnancy and Me: Exploring food insecurity in pregnancy in the UK to inform future Public Health intervention needs.**

**Protocol Version 3.0**

**16-April-2024**

| Chief Investigator: | Dr Nicola Heslehurst |
| --- | --- |
| Sponsor: | Newcastle University |
| Funder: | NIHR School for Public Health Research |
| Sponsor Protocol Number: | 202324 1 Hurley |
| Funder Reference Number: | NIHR 204000: SPHR-CYP-WP2.2-FIP |
| IRAS ID: | 326070 |

**This protocol has regard for the HRA guidance**

1. Signature Page

Food, Pregnancy, and Me study

Protocol version number: 3.0

Protocol date: 16-April-2024

The undersigned confirm that the following protocol has been agreed and accepted and that the Chief Investigator agrees to conduct the study in compliance with the approved protocol and will adhere to the principles outlined in the Declaration of Helsinki, the Sponsor’s SOPs, and other regulatory requirement.

I agree to ensure that the confidential information contained in this document will not be used for any other purpose other than the evaluation or conduct of the investigation without the prior written consent of the Sponsor

I also confirm that I will make the findings of the study publicly available through publication or other dissemination tools without any unnecessary delay and that an honest accurate and transparent account of the study will be given; and that any discrepancies from the study as planned in this protocol will be explained.

| **For and on behalf of the Study Sponsor:** | | |
| --- | --- | --- |
| Signature: |  | Date: 16/ 04/24 |
| Name (please print): Susanne Lewis |  |  |
| Position: Deputy Head of Research |  |  |
| **Chief Investigator:** | | |
| Signature: ...................................................................................................... |  | Date: 16/04/2024 |
| Name: (please print): Nicola Heslehurst |  |  |

#

Contents

[2 Background 1](#_Toc164849645)

[2.1 Food insecurity is a public health priority 1](#_Toc164849646)

[2.2 Food insecurity, health, and well-being 1](#_Toc164849647)

[2.3 UK prevalence of FI, and geographic and socio-demographic inequalities 1](#_Toc164849648)

[2.4 Gender differences in food insecurity prevalence 2](#_Toc164849649)

[2.5 Food insecurity and pregnancy 2](#_Toc164849650)

[3 Rationale and research questions 3](#_Toc164849651)

[3.1 Rationale 3](#_Toc164849652)

[3.2 Aim 3](#_Toc164849653)

[3.3 Objectives 3](#_Toc164849654)

[3.4 Research Questions 4](#_Toc164849655)

[3.5 Outcomes 4](#_Toc164849656)

[4 Theoretical framework 5](#_Toc164849657)

[5 Study design and methods of data collection and data analysis 6](#_Toc164849658)

[6 Sample and recruitment 11](#_Toc164849659)

[6.1 Eligibility Criteria 11](#_Toc164849660)

[6.1.1 Inclusion criteria 11](#_Toc164849661)

[6.1.2 Exclusion criteria 11](#_Toc164849662)

[6.2 Sampling 11](#_Toc164849663)

[6.2.1 Size of sample 11](#_Toc164849664)

[6.2.2 Sampling technique 12](#_Toc164849665)

[6.3 Recruitment 12](#_Toc164849666)

[6.3.1 Consent 13](#_Toc164849667)

[7 Ethical and regulatory considerations 14](#_Toc164849668)

[7.1 Assessment and management of risk 14](#_Toc164849669)

[7.2 Research Ethics Committee (REC) and other Regulatory review & reports 14](#_Toc164849670)

[7.2.1 Regulatory Review & Compliance 14](#_Toc164849671)

[7.2.2 Amendments 14](#_Toc164849672)

[7.2.3 Safeguarding 14](#_Toc164849673)

[7.3 Peer review 17](#_Toc164849674)

[7.4 Patient & Public Involvement 17](#_Toc164849675)

[7.5 Protocol compliance 18](#_Toc164849676)

[7.6 Data protection and patient confidentiality 18](#_Toc164849677)

[7.7 Indemnity 19](#_Toc164849678)

[7.8 Access to the final study dataset 19](#_Toc164849679)

[8 Dissemination policy 20](#_Toc164849680)

[8.1 Dissemination policy 20](#_Toc164849681)

[8.2 Authorship eligibility guidelines 20](#_Toc164849682)

[9 References 22](#_Toc164849683)

[10 Appendices 26](#_Toc164849684)

[10.1 Appendix 1- Required documentation 26](#_Toc164849685)

[10.2 Appendix 2 – Schedule of Procedures 26](#_Toc164849686)

[10.3 Appendix 3 – Amendment History 26](#_Toc164849687)

1. Key Study Contacts

***** Denotes members of the Study Management Group (SMG)

| **Chief Investigator** | |
| --- | --- |
| *Dr Nicola Heslehurst | Senior Lecturer in Maternal Nutrition  Newcastle University  🕿 0191 2083823  🖂 nicola.heslehurst@newcastle.ac.uk |
| **Principal Investigators** | |
| *Christine Moller-Christensen | Research Midwife  Gateshead Health NHS Foundation Trust  🕿 0191 445 2144  🖂 christine.moller-christensen@nhs.net |
| *Nicola Flint | Research Midwife  University Hospitals Coventry and Warwickshire NHS Trust  🕿 02476 964982 Ext: 24982  🖂 nicola.flint@uhcw.nhs.uk |
| **Co-Investigators** | |
| *Professor Heather Brown | Professor of Health Inequalities  Lancaster University |
| *Professor Kate Jolly | Professor of Public Health  University of Birmingham |
| *Dr Kiya Hurley | Senior Research Fellow  University of Birmingham |
| *Professor Judith Rankin | Professor of Maternal and Child Health  Newcastle University |
| *Professor Amelia Lake | Professor of Public Health Nutrition  Teesside University |
| *Dr Steph Scott | Lecturer in Public Health  Newcastle University |
| *Dr Gina Nguyen | Postdoctoral Research Associate  Newcastle University |
| *Dr Kerry Brennan-Tovey | Postdoctoral Research Associate  Newcastle University |
| Dr Zainab Akhter | Postdoctoral Research Associate  Newcastle University |
| Dr Ella Dyer | Postdoctoral Research Associate  Newcastle University |
| *Dr Rachel Loopstra | Senior Lecturer  University of Liverpool |
| *Alice Wiseman | Director of Public Health, Gateshead Council |
| *Angela Baker | Consultant in Public Health, Health Inequalities and Life Chances, Coventry City Council |
| Dr Sushma Acquilla | Independent Consultant in Public Health |
| Emma Gibson | Health Improvement Practitioner, Gateshead Council |
| Harbir Nagra | Programme Manager Public Health, Coventry City Council |
| **Public Representatives and collaborators** | |
| *Dianne Williams | Chief Executive Officer Moat House Community Trust (Registered Community Charity) and Trustee Coventry Feeding Network (Charitable Incorporated Organisation) |
| *Councillor Sonya Dickie | Founder Chair – Felling Food Network; Ward Councillor for Gateshead Council |
| **Sponsor** | |
| Newcastle University | Newcastle University, Newcastle upon Tyne, Tyne and Wear, NE1 7RU  🖂 [sponsorship@newcastle.ac.uk](mailto:sponsorship@newcastle.ac.uk) |
| **Funder** | |
| National Institute for Health and Social Care Research, School for Public Health | William Leech Building, Newcastle University, Newcastle upon Tyne, Tyne and Wear, NE2 4HH  🕿 0191 208 7194  🖂 [sphr@newcastle.ac.uk](mailto:sphr@newcastle.ac.uk) |
| **Study Steering Committee (SSC)** | |
| Professor Maria Bryant (Chair) | Professor of Public Health Nutrition  University of York |
| Dr Dougal Hargreaves | Houston Reader in Paediatrics and Population Health  Imperial College London |
| Dr Zoe Bell | Research Associate  Kings College London |
| Dr Julie Abiyomi | Associate Head of Medicine and Nutrition  Edge Hill University |
| Professor Jane Sandall | Professor of Social Science and Women's Health  Kings College London |
| Ms Courtney Swan | Expert by Lived Experience |
| Dr Charlotte Wilson | Obstetric Consultant  Gateshead Health NHS Foundation Trust |

1. Roles and Responsibilities

Project Management and Oversight:

The Newcastle University is the nominated sponsor of the study. The study will have an externally appointed study steering committee (SSC) that will include a lay member and subject experts. The SSC will meet twice a year or as deemed necessary to monitor study progress. The co-investigator team will form the Study Management Group (SMG) and will meet regularly (2-4 times) per year to oversee the study. Day to day management of the study will be undertaken by the appointed research fellow(s). NH, HB, KJ, and KH will form a senior project management team, which will oversee the day-to-day management undertaken by the project research fellow(s).

The following functions falling under the responsibility of the sponsor will be delegated to Dr Nicola Heslehurst [Chief Investigator]:

- Ethics Committee Opinion (including application for research ethics committee favourable opinion, notification of protocol amendments and end of study, site specific assessment & local approval)
- R&D Approval (including application for global checks, via NIHR CSP)
- Good Clinical Practice (including GCP arrangements, data monitoring, emergency & safety procedures)
- Administration of funding for the study
- The research team is trained in the principles of GCP. This training must be updated at least every 3 years but should be pragmatic to the study.
- Ensure all the necessary employment contracts, honorary research contracts or other access arrangements are in place for all research staff before the study commences.
- Ensure that all employment contracts remain active throughout the study. Where an employee is working under the research passport scheme, continuous access must be maintained and renewal applications should be received by R&D three (3) months in advance of expiry.
- Ensure that, as applicable, arrangements (including staff training and competence) are in place for obtaining informed consent from research participants before research activity is undertaken.
- Ensure that amendments to the protocol or other study documentation are submitted to each Trust R&D for review to confirm that there are no changes to Confirmation and Capacity status of the research. Amendments must also be submitted to the REC and Regulatory Authorities for approval as applicable.
- Notify Trust R&D, REC and Regulatory Authorities as appropriate of changing timelines on a study and of the end of a study.

**Study conduct at site, PI responsibilities:**

- Daily management of their research at their site.
- Ensure Trust-wide Policies and Standard Operating Procedures (SOPs) and/or study-specific SOPs are followed.
- Ensure that the necessary approvals for their research are in place prior to the research commencing including but not limited to:
  - A favourable opinion from a Research Ethics Committee
  - NHS Capacity and Capability from Trust Research & Development (R&D) (NJRO)
  - Approvals from relevant regulatory bodies (e.g., MHRA, NIGB, ARSAC)
- Ensure all essential documents are maintained for their research in the form on an Investigator Site File (ISF)/ Project file.
- Ensure research is conducted in accordance with the approved protocol (unless an Urgent Safety Measure (USM) is required) and that appropriate systems are in place to guarantee version control of documentation e.g., Protocol/abstract, to ensure researchers are working to the correct and most recent version.
- Ensure that each member of their research team is qualified by education, training, and experience for their role in the study.
- Protect the integrity and confidentiality of research data.
- Abide by all appropriate legislation in relation to patient data, including but not limited to:
  - Data Protection Act 2018
  - Mental Capacity Act 2005
- Ensure that an appropriate PI is always named on the study. In cases where an Investigator will be absence from employment (extended sick leave/maternity) or leaves their position and interim or new PI must be nominated and approved by R&D.
- Ensure that all clinical staff have the applicable registrations and licences to practice for the duration of the study, including any revalidation requirements.
- Provide access to all study documents, devices and equipment as required for monitoring, auditing, and inspection purposes.
- Ensure appropriate archiving of their investigator site file/project file at the end of a study.

**Funder’s responsibilities:**

The funders have no role or responsibility for the study design, conduct, data analysis and interpretation, manuscript writing, and dissemination of results. The funders do not control the final decision regarding any of these aspects of the study.

1. STUDY SUMMARY

| Study Title | **Food, Pregnancy and Me: Exploring food insecurity in pregnancy in the UK to inform future Public Health intervention needs** |
| --- | --- |
| Internal ref. no. (or short title) | **Food, Pregnancy and Me** |
| Background | Food insecurity is when someone struggles to afford or access enough food. More than 1 in 6 people in the UK have food insecurity, which means they have skipped meals, have not eaten when hungry, or have not eaten for one full day. This can affect the type and amount of food they eat, and their physical and mental health. Food insecurity has become more common in the UK since the 2008 recession, and we now have record numbers nationally. The highest number of people experiencing food insecurity are in the North East of England. Also, more than 1 in 4 women and children are food insecure. This further increases to almost half of households with three or more children. The UK now faces a cost-of-living crisis and another recession estimated to last at least two years. It is extremely likely the number of people experiencing food insecurity will get even higher.  During pregnancy, the food pregnant people eat is important for both their own and their baby’s health. For example, some vitamins and minerals are essential for the baby’s development. However, research from the USA shows these needs are rarely met when food insecure. This increases the risks to the pregnant person, such as developing depression, and to the baby, such as being born too early. These have lifelong knock-on effects for the health of the mother and their children. It also increases costs for maternity services, wider health services, and society. However, little is known about food insecurity during pregnancy in the UK. For example, how many pregnant people are food insecure, how does this affect their diet quality, their health, and their baby, what are their experiences and what support might help them the most? We need to know this information to help plan support. |
| Study Design | **Work package 1 (WP1):** We will ask 605 pregnant people attending maternity services in Gateshead and Coventry to answer questions on food insecurity, their diet and health. We will also use pseudo-anonymised information that is routinely collected by maternity services during pregnancy. This information will help us identify how many women might need support, and which areas of their own and their baby’s health and well-being most need support. This will help to inform what type of support is needed and which services might need to be involved.  **Work package 2 (WP2):** We will ask 40 people who have completed the questionnaire, and are identified as being food insecure, about how this has impacted them during their pregnancy, and what help and support they might find most useful during and after pregnancy. This will help us get a better understanding of what difficulties they face and how complex these are.  **Work package 3 (WP3):** Using co-production methods with people who have lived experience of food insecurity during pregnancy, and important professional and charity stakeholders, we will develop strategic recommendations for support required to mitigate the impact of food insecurity during pregnancy. This will be informed by the results of WP1 and WP2 which will be compared, contrasted, and combined and discussed with stakeholders. We will then use an economic technique called Expected Value of Sample Information to determine how many people we would need to recruit to evaluate any of the recommendations and the likely costs and benefits of conducting vs not conducting future research in the area. |
| Study Participants | **WP1:** Pregnant people in their third trimester (≥28 weeks gestation) receiving care by maternity services based at Queen Elizabeth Hospital Gateshead or University Hospital Coventry.  **WP2:** Those who took part in WP1 and were identified as food insecure, and expressed an interest in participating in WP2. |
| Planned Size of Sample | **WP1:** 605 pregnant people  **WP2:** Approximately 40 people (20 at each site) |
| Planned Study Period | This research project will last for 20 months. |
| Research Question/Aim(s) | The aim of this research is to identify the levels of food insecurity in pregnancy in the North East and West Midlands, how this impacts on diet and pregnancy health outcomes for pregnant people and their babies, the costs of this to maternity services and wider healthcare, and to explore experiences and support needs.  **Research questions**   1. What is the prevalence of food insecurity in pregnancy and how does it impact on maternal nutrition and pregnancy health outcomes in highly deprived regions in England (WP1)? 2. How do pregnant people experience and negotiate food insecurity and what are their support needs (WP2)? 3. How can public health policy and practice be developed to address food insecurity in pregnancy (WP3a: co-production of strategic recommendations from WP1-2)? 4. What is the Expected Value of Information of the interventions (based on strategic recommendations) (WP3b)? |

1. Funding and support in kind

| **FUNDER(S)** | **FINANCIAL AND NON FINANCIALSUPPORT GIVEN** |
| --- | --- |
| **National Institute for Health and Social Care Research, School for Public Health (SPHR)** | William Leech Building, Newcastle University, Newcastle upon Tyne, Tyne and Wear, NE2 4HH  🕿 0191 208 7194  🖂 [sphr@newcastle.ac.uk](mailto:sphr@newcastle.ac.uk)  **Role:** SPHR has provided the financial support for this study. The funders have no role or responsibility for the study design, conduct, data analysis and interpretation, manuscript writing, and dissemination of results. The funders do not control the final decision regarding any of these aspects of the study. |
| **National Institute for Health and Social Care Research, Applied Research Collaboration: West Midlands (ARC WM) and North East and North Cumbria (ARC NENC)** | ARC West Midlands, University of Warwick Office, Room B146, 1st Floor, Health Sciences, Warwick Medical School, University of Warwick, Coventry, CV4 7AJ  🕿 024 7652 4794  🖂 ARCWM@warwick.ac.uk  ARC North East and North Cumbria, St Nicholas’ Hospital, Jubilee Road, Gosforth, Newcastle Upon Tyne, NE3 3XT  🕿 0191 246 7369  🖂 arcnenc@cntw.nhs.uk  **Role:** ARC WM has provided financial support for this study through funding 0.2FTE of a researcher (KH) to deliver the qualitative aspects of this study in the West Midlands and manage the Public Involvement and Engagement for the region. It has also supported KJ’s time as a co-investigator. ARC NENC has provided supported time for KBT and JR. The funders have no role or responsibility for the study design, conduct, data analysis and interpretation, manuscript writing, and dissemination of results. The funders do not control the final decision regarding any of these aspects of the study. |

| 1. Key words: | Food insecurity  Pregnancy  Observational study  Health economic evaluation |
| --- | --- |

1. Study flow chart

Research midwives identify eligible pregnant people, create a unique participant identification number (PIN), and post a questionnaire pack.

**Work package 1**

Research team contacts those who are identified as food insecure and have stated they are interested in being interviewed to share PIS and consent form to review.

40 food insecure participants are interviewed about their experiences and recommendations for change.

Interviews are transcribed, coded and themes identified.

**Work package 2**

**Work package 3**

University research team removes any identifying information from the questionnaire (e.g., name and address to be sent the thank you voucher) and stores it separately from the questionnaire.

Research team send the PIN of all responding participants to the NHS team

NHS team links the PIN to the maternity record, creates a pseudonymised copy and sends to the University research team.

Quantitative and qualitative analysis of the questionnaire and analysis of associations between food insecurity and health outcomes.

Participants return the questionnaire to the University research team (via post or digitally).

University research team analyses the food insecurity questionnaire to identify food insecure participants.

WP3a: Co-production of strategic recommendations using results from each WP with stakeholders and public contributors.

WP3b: Expected Value of Sample Information (EVSI) modelling to determine if there is a benefit in intervening with the prioritised strategic recommendations.

Outcomes workshop held with stakeholders and the public to discuss WP2 results.

Outcomes workshop held with stakeholders and the public to discuss WP1 results.

**STUDY PROTOCOL**

Food, Pregnancy and Me: Exploring food insecurity in pregnancy in the UK to inform future Public Health intervention needs

# Background

## Food insecurity is a public health priority

Food insecurity (FI) “*exists whenever the availability of nutritionally adequate and safe foods or the ability to acquire acceptable foods in socially acceptable ways is limited or uncertain”* (1)*.*Since the global financial crisis of 2008, FI has become increasingly prevalent in high-income countries, and is usually caused by poverty, unemployment, and low income (2-4). In the UK, the introduction of austerity measures is suggested to be a major contributing factor (5). For example, the introduction of Universal Credit and associated problems, such as payment delays, resulted in increased use of emergency food aid (5, 6). More recently, the COVID-19 pandemic, Russia’s invasion of Ukraine, Brexit, and the increased cost-of-living due to fuel, food, and mortgage prices, have increased the prevalence of FI in the UK (6-11). Although the UK government announced an Energy Price Guarantee, in which average households would not pay more than £2500 per year on their energy bill from October 2022 for two years, this is still a considerable increase from the price cap of £1277 from the previous winter (12). It is highly likely that the cost-of-living crisis, and associated FI, could worsen considerably over the coming years without further intervention.

## Food insecurity, health, and well-being

FI is associated with a nutritionally poor diet, consumption of energy-dense, low quality, sugary, salty, high-fat and processed food, and living in an obesogenic environment; thus increasing the risk of developing chronic diseases such as obesity, type 2 diabetes and cardiovascular disease and poor dental health (13-21). The nutritional quality of emergency food aid is also inadequate, exceeding energy requirements and deficient in micronutrients such as vitamins and iron (22-24). FI can also lead to high levels of stress, anxiety, depression, and sleep difficulties (25-29). There is also stigma associated with FI, including the use of food aid (4, 30), which may lead to delayed help seeking and worsen the severity of FI (31).

## UK prevalence of FI, and geographic and socio-demographic inequalities

The dramatic increase in FI over the past 15 years has resulted in an increase in usage of emergency food aid such as food banks, soup kitchens, and food stamps (2). In the UK, food aid has become normalised with the presence of food banks throughout all areas of the country (2, 4). However, as food aid only eases the symptoms of FI, rather than treating the root cause, little progress has been made to reduce prevalence of FI (2, 3). Recent data show the reverse trend, with record high prevalence in households experiencing FI in 2022 (32). In September 2022, 9.7 million adults (18.4% of households) had experienced moderate/severe FI in the past month, including skipping meals, not eating when hungry or not having eaten for one full day. There was substantial geographic and socio-demographic variation in prevalence. For example, 27.8% prevalence in the North-East of England compared with 14.1% in the South-West. Prevalence increased from 16.0% of households without children, to 25.8% of households with children, and 42.2% of households with ≥3 children. FI was also higher among people with disabilities than without (45.4% vs 13.1%), in households receiving Universal Credit than not (53.8% vs 15.6%), and among Black, Asian, and other minoritised ethnic groups than White (26.9% vs 18.5%). Rising energy costs contributed to FI. Among all households included in the survey, 68.1% were worried about the impact of increasing energy prices on their ability to buy enough food for their household's needs. Households experiencing FI were more likely to cut back their spending on fruit (58.0%) and vegetables (47.7%) compared with those not experiencing FI (12.8% and 7.8% respectively). Additionally, data collected by the Department for Work and Pensions (DWP) on Household Food Security in 2020/2021 (33) highlighted that households where the head of the household was under 25 years old, and single parent families, were most likely to report FI.

## Gender differences in food insecurity prevalence

Households with women and children are most likely to experience FI (32, 34), and single parents in the UK are most likely to use foodbanks; 84% of which are female (6, 10). Women are particularly vulnerable to FI due to working in low-income or part-time jobs, as well as societal expectations for them to be the main carers for children and family members, carry out unpaid housework, and be the main providers of food (35-37). When finances are scarce, women report restricting their own food intake in favour of their children and other household members (38-40), making them more vulnerable to the nutritional impact of FI. There is also a significant association between FI and maternal clinical depression, which may have a bi-directional causal relationship (25, 41, 42).

## Food insecurity and pregnancy

 FI can lead to poor physical and mental health for women during the life course transition to pregnancy (26, 37). There are increased nutritional demands during pregnancy for both maternal and foetal health, making it a critical time for adequate nutrition (37, 43). Fertile women are prone to micronutrient deficiencies and undernourishment generally, which puts them at higher risk of ill-health during times of FI (37). This is particularly relevant for pregnant women as they have elevated nutrient requirements, such as folate and iron, compared with the general population (44). FI during pregnancy can significantly increase the risk of pregnancy complications and adverse pregnancy outcomes, such as gestational diabetes, and preterm birth (18, 37). These have lifelong health implications for women and their children such as increased risk of developing obesity, type 2 diabetes, and cardiovascular disease. FI also adversely affects pregnant women’s and mother’s mental health, including increased risk of depression and anxiety (45). FI affecting maternal nutrition, and deficiencies, also changes in the in-utero environment resulting in epigenetic changes to foetal development (46) which impact on the infant’s life-long health and wellbeing (47, 48). This life course stage is crucial for public health intervention to promote the best start in life to help mitigate some of the environmental exposures to inequalities following birth.

# Rationale and research questions

## Rationale

The research question/aims have been informed by working directly with multiple public health stakeholders to address the evidence-based gaps required for public health decision making. The stakeholders include a wide “public health workforce” which encompasses Local Authority, Maternity Services, and the Voluntary Sector. These stakeholders have been involved in developing the research and are included as co-investigators on the research.

Public and community partnership has been embedded throughout this proposed research, including setting our research aims, designing the research methods, reviewing the plain English summary, and we will continue to work with public and patients with lived experience of FI in pregnancy throughout the conduct, interpretation, and dissemination. The proposed research includes strategies to be inclusive and engage under-represented populations by working with public involvement and engagement (PI&E) members who have lived experience of the intersecting complex poverties that exist alongside FI.

## Aim

To explore the prevalence and health impact of FI in pregnancy among a geographically and ethnically diverse population of pregnant people living in regions of high deprivation in England to develop strategic recommendations for intervention strategies.

## Objectives

1. To quantitatively measure FI in the third trimester of pregnancy to estimate prevalence and its association with socio-demographic characteristics in two highly deprived locations (Gateshead and Coventry) with diverse ethnic populations (Work Package (WP)1).
2. To link pregnancy FI data with routine pregnancy maternal health records to estimate associations between FI (and severity) with maternal and child health (short-term) and model impact on longer-term health (WP1)
3. To quantitatively collect maternal diet quality data in the third trimester to explore associations between FI (including severity) and maternal diet quality (WP1)
4. To qualitatively explore the experiences of people experiencing FI in pregnancy and their support/intervention needs (WP2)
5. To work with a range of stakeholders to use the evidence generated in WP1&2 to co-produce strategic recommendations for how different (local) stakeholders can provide intervention support (including, but not limited to, structural and policy interventions) for FI during and after pregnancy. A-priori identified stakeholders include people who have lived experience of FI in pregnancy, local authorities, Health Determinants Research Collaborations (HDRCs), maternity services, and voluntary organisations; these will be further informed by PI&E and stakeholder engagement activities, and evidence from the research (WP3a).
6. To conduct health economics analysis (Expected Value of Sample Information (EVSI)) based on the strategic recommendations for intervention support to help decision makers plan future interventions (WP3b)

## Research Questions

1. What is the prevalence of food insecurity in pregnancy and how does it impact on maternal nutrition and pregnancy health outcomes in highly deprived regions in England (WP1)?
2. How do pregnant people experience and negotiate FI and what are their support needs (WP 2)?
3. How can public health policy and practice be developed to address FI in pregnancy (WP3a)?
4. What is the Expected Value of Information of the interventions (using data from WP1-3a) (WP3b)?

## Outcomes

**WP1 Outcomes:** Establishing FI in pregnancy prevalence estimates, and associations with maternal and child health, to inform future public health intervention planning.

**Primary health outcomes:**

- Maternal: antenatal depression
- Child: preterm delivery (<37 weeks)

**Secondary health outcomes:**

- Maternal:
  - gestational diabetes
  - preeclampsia
  - pregnancy induced hypertension
  - mode of delivery
  - induction
  - length of stay in maternity unit
  - maternal diet/nutrition
- Child:
  - birthweight
  - large- and small-for gestational age
  - breastfeeding initiated/at discharge from maternity services
  - admission to special care baby units (and length of stay)
  - Apgar score

**WP2 outcomes:**

- An understanding of the experiences and support needs of food insecure pregnant people

**WP3 outcomes:**

- WP3a: Prioritised strategic recommendations
- WP3b: EVSI economic modelling

# Theoretical framework

This research will employ a mixed methods approach, consisting of concurrent qualitative and quantitative data collection and triangulation. These types of data are underpinned by different research paradigms. For example, quantitative data is usually underpinned by positivism, whereby there is an assumption that there is a ‘real’ world that can be measured. Whereas, qualitative data employs an interpretivism model, which assumes we all experience the world differently (49). Each type of data addresses different questions and delivers different types of answers, however purposefully integrating these outputs can enrich the evidence and provide a deeper, more diverse understanding of the phenomena being studied (50).

We will utilise our qualitative data to develop a deeper understanding of the experiences and impacts of being food insecure during pregnancy, experienced by those identified as food insecure through the quantitative data collection. These data will be collected in parallel and an interactive approach may be used whereby the data collection and analysis can drive changes in the data collection procedures but data is integrated after each source of data is analysed (50).

This approach is appropriate to understand the barriers and facilitators experienced and perceived by the those who experience food insecurity in pregnancy and develop strategic recommendations for support.

# Study design and methods of data collection and data analysis

The planned research is a sequential mixed methods study with data integration from WP1 (quantitative) and WP2 (qualitative), and PI&E and stakeholder engagement perspectives, to inform strategic recommendations which will be the basis of WP3 (co-production and health economics).

**WP1: Measurement of FI and diet in pregnancy, and health data linkage**

***Study design and population****:* This will be a prospective quantitative study with routine data linkage. The population will be pregnant people accessing maternity services in the North-East (Gateshead) and West-Midlands (Coventry) regions. The North-East region has the highest prevalence of FI and child poverty in the UK. In Coventry, 26% of Lower-layer Super Output Areas are in the top fifth most deprived in England (compared to 20% across England overall) and it is ranked particularly high for income deprivation affecting children. Gateshead NHS Trust has an annual birth rate of 1800 and Coventry NHS Trust of 6000.

Pregnant people in their third trimester will be recruited via a questionnaire pack sent to them directly by the maternity service in charge of their care (i.e., either Queen Elizabeth Hospital Gateshead or University Hospital Coventry). Accessing pregnant people through maternity services will ensure all pregnant people are given the option to participate. The questionnaire pack will include a cover letter, the PIS, the questionnaire, a self-addressed envelope, a pen, and the option to complete the questionnaire digitally to facilitate convenient completion. If completed on paper, the hard copy will be posted directly to the research team at Newcastle University.

***Data measures and collection methods:***

Questionnaires: will include data that are not routinely recorded by maternity services. Data linkage with routine maternal and child health records will take place following the delivery of the baby and discharge from maternity care, using maternal and infant NHS number. All data will be anonymised using unique participant IDs before sharing with research team members outside of NHS Trusts.

- *Food Insecurity:* FI will be prospectively measured using the USDA Food Security Survey Module (USDA FSSM) (51) and the complementary measures of stability, availability and utilisation developed by Calloway et al (2023) (52). The USDA FSSM captures varying levels of severity of FI, initially by the uncertainty and anxiety regarding the lack of food access, followed by compromises in dietary quality (i.e., eating less balanced diets) as the situation worsens. With increasing the level of FI severity, the quantity of food consumed decreases as portion sizes are reduced or meals are skipped. At the most severe stage of FI, households could experience absolute food deprivation (i.e., individuals’ not eating at all for a day or experiencing hunger). The full model of the USDA FSSM includes 18 questions, of which 10 assess the general experience of adults in the household. The remaining 8 questions are asked only if there are any children under 18 years living in the household (51). This questionnaire was developed in the USA and has been validated and used in different countries/contexts, including the UK (53). An additional ‘three pillars of food insecurity’ will also be asked to assess food insecurity stability, perceived limited availability, and barriers to utilisation of food (52). Additional questions related to food coping strategies specific to pregnancy will be added to explore further aspects of the FI experience among pregnant people (54). By assessing different food coping strategies, for example, the food they choose when having financial constraints, use of food banks, or selling possessions to help pay for food, will bring further context to the FI experience and impact on social wellbeing. Participants will be asked to complete the food insecurity questionnaire based on their experiences during their pregnancy, and also reflecting on their preconception experiences.
- *Socio-demographics:* Routine maternity data does not include many of the individual level socio-demographics that would be relevant to FI context. We will include questions relating to education, employment, receipt of benefits (including those specific to pregnancy such as healthy start vouchers, and more general benefits such as universal credit, disability), income, support networks and household occupancy. We will explore additional socio-demographics that may be important with the PI&E group before finalising this section of the questionnaire.
- *Diet quality:* Early PI&E work has explored the acceptability and feasibility of a range of dietary measurement methods in this population. We acknowledge the complexities of measuring diet quality in this population, particularly in relation to pregnancy and cultural diversity. The tool we will use is an adapted version of the Brief Diet Quality Assessment Tool (BDQAT) (55). Adaptations to the original tool were made, in consultation with public involvement representatives, to ensure it was acceptable to a variety of people, including those who are on a low-income, from a minoritised community, or pregnant. Adaptations included, adding sausages and burgers (animal source unspecified) to item 1, removing the alcohol category, add adding example food items that are more commonly consumed in several minoritised communities in the UK (e.g., different types of bread (lavash, kulcha, chapattis) and sweet foods (jalebi, halwa, sweet valenki). We will include open ended questions to enable participants to describe the impact of food insecurity on their diet during pregnancy and in the preconception period, and whether they have had to make any changes.
- *Edinburgh Postnatal Depression Scale (EPDS)* (56, 57)*:* Maternity services routinely screen for indicators of depression using the Whooley screening questions, and only conduct the EPDS where there is an indication of depression. As maternal depression is the primary health outcome for this study, we will include the EPDS in the questionnaire to ensure this is completed for all participants. The EPDS consists of 10 questions exploring how pregnant people have been feeling over the previous seven days. The maximum score is 30. A score of 10 or more indicates possible depression, and scores above 13 indicate depressive illness of varying severity.

Missing data: In instances where returned questionnaires contain missing data, the University Research Team will attempt to retrieve this data from the participant directly. Participants will only be contacted in instances where participants have provided their contact details and given permission to be contacted for this reason. The University Research Team will make up to three attempts to contact the participant to retrieve the missing data using the contact details provided.

Data linkage: Data collected via questionnaires will be linked with routine maternity records for the mother and their baby. Data will be exported from the electronic patient records and linked using NHS numbers and unique participant identification numbers for each participant. Where participants live outside of Coventry or Gateshead, this may involve the NHS team contacting the local NHS Trust for the information required. Routine data to be linked include:

- Maternal and child health outcomes listed in Section 3.4.
- Socio-demographic and clinical variables: maternal age, parity, BMI, ethnic group, deprivation score (using postcode and index of multiple deprivation (IMD) scores), alcohol, illicit drugs, employment, benefits status, relevant previous medical history (e.g., history of depression, previous pregnancy health such as low birth weight).

***Data storage and transfer:***  All paper questionnaires will be stored in a locked fire-resistant cabinet at Newcastle University and only accessed by the research team. Online questionnaire data will be collected via an online survey software which is ISO 27001 and GDPR compliant. To allow us to complete the analysis, all anonymised data will be transferred to a secure server at Newcastle University and stored in accordance with GDPR and the Data Protection Act 2018. Linked pseudo-anonymised data securely transferred from the NHS maternity record will be matched to the questionnaire data using the PIN and stored together with the pseudo-anonymised questionnaire data. All personal information will be securely stored separately from the research data. Personal information for Coventry residents, who provide their contact information, and are eligible to take part in WP2, will be transferred to University of Birmingham via a secure, encrypted transfer system and stored on their secure server only accessible to the research team. Research data will be stored in a suitable repository for ten years after the study end date.

***Data analysis strategy:*** All analyses for WP1 will be carried out in STATA v.17.

- *FI prevalence:* Following methodology proposed by the USDA we will calculate the prevalence of FI in pregnancy, which includes household food insecurity. This includes estimates of overall FI, as well as sub-groups of marginal, moderate, low and very low food security. Logistic regression will explore socio-demographic factors associated with FI in pregnancy, including IMD score, ethnic group, maternal age, employment, benefits, income, education, parity, and household occupancy. We will also explore socio-demographic patterns to identify any population groups particularly vulnerable to FI in pregnancy.
- *Dietary assessment:* We will estimate the associations between FI and maternal diet quality score using regression analysis. Open ended questions will explore impact of food insecurity in relation to preconception and pregnancy diets (e.g., dietary changes made).
- *Pregnancy outcomes:* Regression analysis will be used to explore associations between FI and health outcomes. Odds ratios and 95% confidence intervals will estimate associations and multivariable models will be developed to account for socio-demographic and clinical factors related to both FI and health outcomes. We will use findings from the literature to estimate the potential long-term outcomes of FI during pregnancy.

**WP2: Qualitative research**

***Study design and population:*** This qualitative semi-structured interview study will be conducted in both research sites (Gateshead and Coventry).

Questionnaire respondents who are identified as food insecure based on their questionnaire responses, have provided contact details for the purpose of discussing their experiences during their pregnancy, and are within the study sampling frame (see section 6.2.2) will be contacted by the research team. Interviews will take place in-person and using Zoom, according to the needs of the project and participants.

***Data collection methods:*** Qualitative semi-structured interviews will be conducted with people who are FI and are pregnant or have had a baby in the last 12 months. Interviews will explore their experiences of FI during pregnancy, and what support (including food access support) they would have found most useful during and after pregnancy. Interviewees will be actively encouraged to consider support measures that could be implemented locally beyond/in addition to monetary support, and any potential spillover effect to the wider household. All interviews will use a topic guide developed iteratively across the study. The topic guide was initially informed by the literature and discussions with PI&E members with direct experience of FI in pregnancy. We will review the questionnaire data to identify if any issues are raised by the questionnaire that warrant further qualitative exploration. Whilst it is anticipated that interviews will take place in-person, we are cognisant that remote methods can offer strengths including more flexible scheduling and reduced travel costs to both participants and researchers. Therefore, wherever possible, we will flex according to the needs of the project and participants. All researchers conducting interviews will ensure participants are aware that where there is a safeguarding concern, information may be passed to the appropriate services to ensure the safety of the participant or others. We will endeavour to arrange Interviews on Monday-Thursday to ensure any referrals can be made promptly. Any safeguarding issues identified during the research will be reported to the appropriate safeguarding team (see section 7.2.3), Chief Investigator and Sponsor.

**Data storage and transfer:**  All paper consent forms will be stored in a locked fire-resistant cabinet at Newcastle University and only accessed by the research team. All consent forms collected by the University of Birmingham for Coventry participants will be posted to Newcastle University for storage. Audio-files of interviews will be stored on a secure server at Newcastle University and stored in accordance with GDPR and the Data Protection Act 2018. The audio-files of any interviews conducted by University of Birmingham will be transferred to Newcastle University using a secure, encrypted online transfer. To allow us to complete the analysis, audio-files will be transcribed and all personal data (including any parental names, baby names or midwife names) will be removed from the transcripts prior to analysis. The recordings will be destroyed once the results of the study have been published and all other research data will be stored in a secure repository for ten years after the study end date.

***Data analysis strategy:*** All data will be analysed using reflexive thematic analysis. Interview data will be imported into Nvivo 11 (QSR International, Melbourne, Australia). Transcripts will be coded line-by-line and then systematically indexed into data tables to generate detailed descriptive themes. These descriptive themes will be compared to identify patterns, similarities and differences in the data, and relationships between them elaborated, to generate analytical themes, and a consistent interpretation of the whole dataset. Coding and analysis will be undertaken by researchers at Newcastle University and the University of Birmingham. Themes will be discussed and challenged at regular project meetings, using a process defined as pragmatic double coding. All participants will receive a study information leaflet and provide informed consent; confidentiality and anonymity will be assured. Where interviews are conducted in-person, written consent will be requested. Where interviews are conducted online, consent will be recorded verbally via the research reading the consent aloud and the participant confirming their consent to each statement. Transcripts of the recorded consent will be stored in lieu of written consent forms. It is anticipated that all interviews will last approximately one hour; as a thank you for their time, interviewees will receive a £25 voucher.

**WP3a: Co-development of strategic recommendations from WP1 and 2**

The initial translation of research into strategic recommendations will be carried out for each WP separately and discussed with PI&E and stakeholders. PI&E and stakeholder engagement activities following the analysis of WP1 and WP2 will be part of a larger co-development process to translate the findings of these WPs into strategic recommendations for future intervention. Drawing on the Lundy model (2007), as well as work from Dewa et al. (2021), Smithson et al. (2021) and Smith et al. (2022), we will present back findings from WP1 and WP2, allowing PI&E members and stakeholders to discuss, challenge and revise preliminary themes (58-61). After each session, the research team will take away reflections and recordings and, at the beginning of each subsequent session, spend time discussing/recapping these reflections.

The final stage of drawing together the strategic recommendations will be the point at which the integration occurs. We will use a convergence coding matrix (62, 63) to integrate strategic recommendations arising from WP1 and WP2 (and their associated PI&E and stakeholder engagement sessions). This process will involve actively searching and comparing the data and for any pattern and grouping these based on similarity of concept. We will search for agreement and disagreement between the strategic recommendations from WP1 and WP2, defined as:

- Convergence: where findings directly agree
- Complementarity: findings offer complimentary information on the same issue
- Dissonance: findings appear to contradict one another
- Silence: themes arising from one component study but not others

The results of the convergence coding matrix, including any agreement and disagreement between WPs, will be discussed during a co-development workshop, along with any prioritisation of recommendations. This event will provide space to review, critically discuss, revise, and finalise the draft recommendations. The finalised recommendations will be used in WP3b for the health economics analysis. We will also draw on NIHR guidance on co-creation, specifically the key principles around sharing of power, including all perspectives and skills, respecting and valuing the knowledge of those working together on the research, reciprocity, and building and maintaining relationships.

**WP3b: Health Economics**

***Health economics outcomes****:* The Expected Value of Sample Information (EVSI) measures the ‘expected reduction in expected loss’ from a given intervention. At the population level this can be expressed in terms of quality adjusted life years (QALYs). The difference between the EVSI and the cost of research is the expected net benefit of sampling (ENBS) (64).

The EVSI will be estimated using regression-based methods. First, we will start with probabilistic sensitivity analysis to model the current level of uncertainty based upon literature on adverse pregnancy outcomes associated with FI, our findings, and expert opinion. Next, we will compare an innovative intervention to reduce FI compared to standard care (i.e., the strategic recommendations developed using data from WP1-2, PI&E and stakeholder engagement sessions, and the co-development event). The output of this model is the incremental net benefit (INB) - the difference between the net benefit of the innovative intervention and standard care. If this is greater than zero it is better to intervene (65). When estimating the EVSI, we will account for expectations regarding the imperfect implementation of any of the identified interventions. We will employ a novel method, the moment matching method (65-67), so that the probability of whether a given intervention is cost-effective can be estimated. From this, the implementation adjusted EVSI can be computed. This methodology permits the efficient estimation of the implementation adjusted EVSI, based upon realistic model structures and trial designs (66). A societal perspective will be used for societal perspective will be used in the EVSI.  These findings will provide important evidence for local policy makers and community groups about the cost-benefit of implementing any support strategies identified through co-production and provide the basis for a future intervention evaluation bid going forward.

# Sample and recruitment

## Eligibility Criteria

### Inclusion criteria

For WP1, all pregnant people (age 16 years or above) who register for maternity services based at Queen Elizabeth Hospital Gateshead or University Hospital Coventry are eligible for inclusion once they reach their third trimester of a viable pregnancy (28 weeks gestation).

For WP2, participants must be:

- 16 years old or above.
- Resident in the UK.
- Pregnant OR with 12 months post-partum.
- Be receiving OR have received care in their third trimester by maternity services based at Queen Elizabeth Hospital Gateshead or University Hospital Coventry.
- Identified as food insecure by the research team.

### Exclusion criteria

For WP1, participants must not be:

- Under 16 years old.
- Non-UK resident.
- Receiving care by maternity services other than those based at Queen Elizabeth Hospital Gateshead or University Hospital Coventry.
- Less than 28 weeks gestation or post-partum.

For WP2, participants must not be:

- Under 16 years old.
- Non-UK resident.
- Unable/unwilling to give informed consent to participate.
- Receiving/have received care by maternity services other than those based at Queen Elizabeth Hospital Gateshead or University Hospital Coventry in their most recent pregnancy.
- Less than 28 weeks gestation.
- More than 12 months post-partum.
- Identified as food secure by the research team.

## Sampling

### Size of sample

**WP1:** Food insecurity in pregnancy is an unexplored area in the UK. The novelty of this study means that we do not have accurate prevalence data on which to base sample size calculations, and therefore this study is exploratory. We will estimate prevalence in two maternity units as part of this research that can inform future studies. The data we have used to inform the sample size calculations is based on prevalence estimates of up to 30% for food insecurity (extrapolated from UK general population estimates which use different measurement tool for food insecurity than in our study); Gateshead maternity services having 1800 births per year, Coventry maternity services having 6000 births per year; UK prevalence of antenatal depression of 10%; and for preterm delivery 7-8%. Based on these estimates, and a confidence level of 95%, margin of error of 5%, and estimated attrition rate of 4% (based on similar observational research in maternity services), a sample size of 605 pregnant people would be needed.

**WP2:** Following recommendations for pragmatic assumptions around sample size (68), it is anticipated that approximately 40 people will be interviewed (20 per geographical site). Sample size will be guided by the breadth and focus of the research questions; the demands placed on participants; the depth of data likely to be generated; pragmatic constraints; and the analytic goals and purpose of the overall project.

### Sampling technique

**WP1:** A member of the maternity research team at the recruiting hospitals will provide recruitment packs containing a cover letter, translation page, participant information sheet (PIS), questionnaire, pen, self-addressed envelope, and support leaflet to eligible pregnant people entering their third trimester. There will also be a QR code to enable the questionnaire to be completed online. The rationale for this sampling technique is to be inclusive of all adult pregnant people to derive information on the prevalence of food insecurity in this population.

**WP2:** The sample for the qualitative element of this study will be purposively sampled from the respondents to the questionnaire in WP1 according to their FI status, geographical region, socio-economic status, and ethnic group. As participants will be asked to indicate their income and benefit status within the survey, it is anticipated that socio-economic status will be measured according to these indicators. Where this information is not available, IMD score derived from postcode data will be used as a proxy.

## Recruitment

The recruitment strategy has been informed by PI&E discussions and co-investigators who have vast experience of recruitment to pregnancy research. Participants will receive a £20 voucher for completion and return of their questionnaire and a £25 voucher for completion of an interview. This approach will also maximise inclusivity for the target population, as some experiencing FI might also be experiencing digital exclusion and might have financial barriers to attending hospital-based maternity appointments.

**WP1:** Recruitment of this study population may be challenging; therefore, a flexible approach will be adopted. Recruitment option 1 (listed below) is the primary method. Options 2 and 3 will be employed only if recruitment is not sufficient through option 1; however, ethical approval will be sought for all recruitment methods to enable them to be enacted swiftly if required. Recruitment rates will be monitored regularly and, where issues arise, will be discussed with the study management group (SMG) and study steering committee (SSC) to determine if supplementary recruitment methods should be employed.

Recruitment options:

1. Postal questionnaires sent directly to those in their third trimester of pregnancy (28-40 weeks gestation)
2. Maternity research team in the NHS Trusts will conduct face-to-face recruitment for those attending hospital-based appointments.
3. Face-to-face recruitment from community midwife clinics located in areas where under-represented groups receive their care (e.g., areas of highest deprivation or geographies with high case load of minoritised ethnic groups if these populations are under-represented in the questionnaire respondents). If needed, this will be conducted by staff with Good Clinical Practice and Informed consent training, for example staff from the Clinical Research Network’s Direct Delivery Team.

To enhance recruitment, the study will also be promoted in maternity unit waiting areas via posters and targeted reminders sent through NHS communication routes (e.g., text messages or maternity app push notifications). A short video will also be produced based on the information contained within the Participant Information Sheet. The script for this video will be developed with involvement from experts by lived experience.

We will monitor representativeness of the recruited participants socio-demographics compared with the background pregnant population at Gateshead and Coventry maternity services. The research midwives will export 1-year of routinely collected socio-demographic data for women delivering at their sites including maternal age, deprivation status (Index of Multiple Deprivation (IMD) score calculated from the participant postcode), ethnic group, parity, and body mass index. The maternity research team will replace date of birth with age and postcode with IMD score prior to transferring to the research team. Any under-representation will be discussed with the steering committee to identify if we need to implement recruitment option 3. If any further changes to the recruitment strategy are needed to ensure it is inclusive, then these will be subject to an ethics substantial amendment.

**WP2:** Participants who provide contact information, and are within the sampling frame, will be sent a cover letter, PIS, and consent form for WP2. A follow-up call will be made to give the participants the opportunity to ask questions about the research and organise the most convenient time/place for the interview to take place.

### Consent

For WP1, participants will receive the PIS, locality specific support leaflet (containing information on local food aid, financial and mental health support services), and a cover letter in a pack together with the questionnaire. Based on advice from our public contributors, the cover letter will be succinct, but include the key information needed to decide on participation and a statement about how to access support for participation (translation/interpretation or telephone-assisted questionnaire completion). The cover letter will signpost prospective participants to the enclosed PIS for further information (e.g., on data storage). There will also be contact information available on the PIS for participants who wish to speak to a member of the research team. Return of the questionnaire is considered implicit consent for inclusion in WP1.

For WP2, where interviews are in person, informed written consent will be taken prior to the interview taking place. Where interviews are online, audio-recorded informed consent will be taken verbally at the start of the interview, and stored separately to the remaining transcript, in lieu of a consent form.

# Ethical and regulatory considerations

## Assessment and management of risk

The risk for WP1 of this study is minimal. However, some potential participants may find the subject matter distressing. It will be reiterated in all study information that participation is voluntary. There will also be a support leaflet included in the questionnaire pack to signpost potential participants to support services offered in their local area. For participants who score highly for suicidal ideation on the EPDS within the questionnaire, there will be a robust, locality specific Standard Operating Procedure (SOP) in place to ensure notification of appropriate care services (e.g., process outlines in **Figure 1**).

For WP2, there is the risk that the interviewer may receive information that constitutes a safeguarding issue, either for the interviewee or others. There will be a robust, locality specific safeguarding policy in place that all researchers will be obliged to follow. Researchers will have access to specific information for support service in the locality and contact details for referrals where necessary (see section 7.2.3). All researchers conducting interviews will ensure participants are aware that where there is a safeguarding concern, information will be passed to the appropriate services to ensure the safety of the participant or others. Where interviews are conducted in person, researchers will also follow their employer’s lone working policy.

## Research Ethics Committee (REC) and other Regulatory review & reports

This is a low risk, observational survey, and qualitative interview study. Caldicott, NHS Research Ethics Committee and Health Research Authority Approval and will be in place before the study begins. The study sponsor is Newcastle University.

### Regulatory Review & Compliance

Before the study, a favourable UK Health Departments Research Ethics Service (NHS REC) approval and NHS research and development (R&D) approval to conduct the study will be sought. All correspondence with the NHS REC will be retained.

The end of study is the date of the final visit of the last participant. The Chief Investigator will notify the REC of the end of the study. An annual progress report (APR) will be submitted to the REC within 30 days of the anniversary date on which the favourable opinion was given, and annually until the study is declared ended. If the study is ended prematurely, the Chief Investigator will notify the REC, including the reasons for the premature termination. Within one year after the end of the study, the Chief Investigator will submit a final report with the results, including any publications/abstracts, to the REC.

### Amendments

The Chief Investigator, alongside the SMG, will decide whether an amendment meets the threshold for a substantive change to the protocol. Substantial amendments will be submitted to the NHS REC via IRAS for approval and will not be implemented until further approval has been granted by the NHS REC and the relevant NHS R&D departments. All amendments will be tracked in the relevant section of this protocol.

### Safeguarding

Given the sensitivity associated with the unravelling of emotions surrounding food insecurity, the research team is mindful of this potentially vulnerable group of pregnant people. Voluntary participation, the right to ask any questions, and the right to decline participation at any time, will be emphasised during the data collection. Locally tailored signposting to support services will be included in the questionnaire pack and given to participants again after an interview. Participants will be reminded that where the research team has safeguarding concerns (the participant is a danger to themselves or others), the researcher may have to break confidentiality to ensure their safety. Researcher will follow the process outlined in Figure 1.

**Figure 1**: Process for reporting safeguarding concerns

## Peer review

Peer review was completed by the independent Research Review Panel and Executive Group of the National Institute for Health and Social Care Research (NIHR) School for Public Health Research (SPHR). The research review panel consists of independent public health academics, policy, practice and public members. The Executive Group also contains public health specialists who offer expert opinion.

## Patient & Public Involvement

Public contributors have been involved since the conception of this research project. PI&E sessions were carried out with women with lived experience of FI during pregnancy and a facilitator from the Poverty Truth Commission in Gateshead. The sessions focussed on the acceptability and design of the research, recruitment, participant information, questionnaire design and plans for PI&E within the research going forward.

A summary of some of the key points discussed are:

- The incentive of a £20 voucher is essential for questionnaire completion.
- Having online and paper-based versions of the questionnaire is needed.
- Have an option for someone to explain the questionnaire/research in person (e.g., contact details on the questionnaire for the research team) and the need for a succinct cover letter.
- Include a pen/SAE in the questionnaire pack and ask community midwives to promote the research.
- Importance of women knowing who will access their research data (e.g., important to know that social services will not access their data).
- Having the option of someone helping them to complete the questionnaire (e.g., member of the research team) to be inclusive for people with specific disabilities or low levels of literacy.
- Challenges with diet data collection in this population:
  - Fluctuation in diet day to day/throughout the month.
  - Honesty completing diet questionnaire if they haven’t eaten anything (e.g., potential for participants to lie if they thought social services would find out they were not managing and what the implications of this would be).
  - The need to have paper-based diet data collection (i.e., there are lots of online tools, but they wouldn’t work in this population/would exclude people with severe FI/poverty).
  - To also include questions on where they get/eat food (e.g., formal and informal support networks); fuel costs for cooking food; use of food banks and warm spaces; amount of money spent on food monthly (and number of people in household).

The Plain English summary was also reviewed by public contributors from the NIHR Research Design Service North East and North Cumbria consumer panel for this project. Overall, they articulated that this provided a clear summary of planned research, which they felt sounded incredibly valuable and an important research topic.

We will continue to work in partnership with pregnant women and Poverty Truth in Gateshead and meet regularly as the project progresses. We will also develop relationships with other Voluntary, Community and Social Enterprise (VCSE) organisations to facilitate PI&E activities in Gateshead and Coventry. Stakeholder engagement sessions have also been planned at key points to inform the planned research, as well as interpret the findings and support the co-development of strategic recommendations for FI intervention and support. This will enabling ongoing input, consultation, and feedback.

## Protocol compliance

Occasionally protocol deviations may occur. In the event of a deviation, the sites will ensure adequate documentation and immediate reporting to the Sponsor and Chief Investigator. Deviations from the protocol which are found to frequently recur are not acceptable, will require immediate action and could potentially be classified as a serious breach.

## Data protection and patient confidentiality

All investigators and study site staff must comply with the requirements of the Data Protection Act 1998 with regards to the collection, storage, processing, and disclosure of personal information and will uphold the Act’s core principles. Participant identifiable information will be handled in line with GDPR 2018 principles. Throughout the study Newcastle University data protection policies and procedures will be followed. All data will be kept strictly confidential. Initial data collection and storage of questionnaire data will be via Online Surveys and data will be downloaded and stored on the Newcastle University secure server. All participants will be given a unique identifier (participant identification number (PIN)).

No person within the study team will have sight of both the questionnaire data and identifiable information from the maternity record. A research midwife at each hospital will record the PIN and NHS number for each questionnaire distributed. The questionnaire data will be returned by the participants directly to the research team at Newcastle University, who will separate the questionnaire from any identifying information provided (e.g., for the purposes of contact for WP2, sending of thank you voucher, or dissemination of study findings). Following completion of all other data collection (i.e., from routine maternity records), anonymised electronic research data will be transferred to a Newcastle University secure server for analysis. The Newcastle University research team will then send only the PIN to the research midwife, who will return the required, pseudo-anonymised data from the maternity record via a secure, encrypted transfer. See **Error! Reference source not found.** for the flow of data between the NHS teams and the research team. No personal identifying information will be presented in the study outputs.

Personal data for Coventry participants provided for the purpose of recruitment for WP2 will be transferred to the University of Birmingham via a secure, encrypted online transfer and stored securely at the University of Birmingham. Once interviews have been completed, audio-recordings of the interview will be transferred back to Newcastle University via secure, encrypted transfer to be transcribed. The recordings will be destroyed after the results of the study have been published.

Figure 2: Flow of data through WP1

**To keep your information anonymous, the Research Team will:**

**The NHS team will not have access to any of your questionnaire data. They will:**

Remove any identifying information that you put on your questionnaire (e.g., your name and address to be sent your voucher) and stores it away from your questionnaire.

Send your participant identification number (PIN) to the hospital team. When you have had your baby, the research team will ask the hospital team to send us some information that we need for the research from your routine maternity records, like whether you developed diabetes during pregnancy and how much your baby weighed when they were born.

Use your PIN to match the information the NHS team send with your questionnaire responses to use for the research.

Use your PIN to find the information we need for the research from your maternity records.

Remove any identifying information (like your NHS number or name) to make it anonymous.

Return the information to the research team with your PIN.

Assign you a unique participant identification number (PIN) for this research study and send you the questionnaire pack.

## Indemnity

The University has in force a Public Liability Policy and/or Clinical Trials policy which provides cover for claims for "negligent harm" and the activities here are included within that coverage. As all of the research participants are NHS patients or will be recruited at NHS sites then the NHS indemnity scheme will apply. No provision has been made for indemnity in the event of a claim for non-negligent harm.

## Access to the final study dataset

Data will be stored securely with restricted access for a period of three years after the end of the study. Any identifiable data and anonymised research data will be stored by Newcastle University on a secure server.

# Dissemination policy

## Dissemination policy

The detail in this section relates to our preliminary plans for dissemination, communication, and pathways to impact. We will develop a communication, dissemination and impact plan with our PI&E collaborators and wider stakeholders as a strategy for being responsive to a variety of perspectives on how to make science open and reach the intended audiences. This will include a mix of academic outputs, non-academic outputs, and knowledge exchange activities.

**Academic publications:** We plan to have five journal articles using reporting outputs from this study. All will be published open access. These will be:

1. FI prevalence (WP1);
2. FI impact on maternal nutrition and diet quality (WP1);
3. FI impact on maternal and child health outcomes (WP1);
4. economic analysis (WP3);
5. qualitative research with women experiencing FI (WP2).

**Conferences and meetings:** We will submit abstracts for presentation at UK and international conferences. We will also seek local opportunities to engage with stakeholders and disseminate research findings, for example at the Health and Wellbeing Boards in Gateshead and Coventry, local council portfolio boards and public health team meetings. We will continue to work with Gateshead and Coventry stakeholders and co-investigators to identify additional opportunities and will be responsive to opportunities as they arise.

**Non-academic outputs:** We will work with our PI&E and stakeholder groups to develop a public dissemination and impact plan. We will tailor the outputs to the needs of the different target populations, which will also be developed throughout the course of the research, for example utilising research briefs, policy briefs, media coverage and stakeholder and participant communication to achieve this goal. Initial stakeholders/target audiences include pregnant people and their families, public, maternity and health visiting services and health professionals, public health organisations and professionals including HDRCs, policy makers, commissioners and VCSEs. We will also work with the NIHR School for Public Health Research Knowledge Exchange broker to identify further opportunities to disseminate to a wide variety of audiences.

Dissemination of the whole programme of work will focus on maximising its impact on the delivery of routine care for pregnant people experiencing food insecurity. The target audience for this work includes:

- health professionals/organisations (e.g., Royal College of Midwives, Institute of Health Visiting)
- pregnant people and their families
- maternity managers and commissioners of services
- national and international policy makers and decision makers (e.g., NICE, CMO’s, WHO)
- wider public and media
- third sector (e.g., Tommy’s the Baby Charity, Food Foundation, food bank networks)
- academics

## Authorship eligibility guidelines

Results of this study will be submitted for publication in peer reviewed journals. The manuscript will be prepared by the SMG and authorship will be determined by mutual agreement in accordance with the International Committee of Medical Journal Editors recommendations. A Plain English summary of the results will also be published.

Any publications and presentations prepared by Investigators will be reviewed and approved by the SMG. Manuscripts will be submitted to the SMG in a timely fashion and in advance of being submitted for publication, to allow time for review and resolution of any outstanding issues.

Funders will be acknowledged in all publications arising from the study in accordance with their terms and conditions. For example, using the following funding acknowledgement and disclaimer in all publications and research outputs:

“This study is funded by the National Institute for Health and Care Research (NIHR) School for Public Health Research (SPHR) (Grant Reference Number NIHR 204000) and supported by the National Institute for Health and Care Research (NIHR) Applied Research Collaborations in the West Midlands (ARC WM) and North East and North Cumbria (ARC NENC). The views expressed are those of the author(s) and not necessarily those of the NIHR or the Department of Health and Social Care.”

Participants will receive a summary of the study results where they have provided contact details for this purpose.

# References

1. Anderson. Core indicators of nutritional state for difficult-to-sample populations. J Nutr. 1990;120 Suppl 11:1559-600.

2. Davis O, Geiger BB. Did Food Insecurity rise across Europe after the 2008 Crisis? An analysis across welfare regimes. Social Policy and Society. 2017;16(3):343-60.

3. Lambie-Mumford H, Green MA. Austerity, welfare reform and the rising use of food banks by children in England and Wales. Area. 2017;49(3):273-9.

4. Purdam K, Garratt EA, Esmail A. Hungry? Food Insecurity, Social Stigma and Embarrassment in the UK. Sociology. 2016;50(6):1072-88.

5. Yau A, Singh-Lalli H, Forde H, Keeble M, White M, Adams J. Newspaper coverage of food insecurity in UK, 2016–2019: a multi-method analysis. BMC Public Health. 2021;21(1):1201.

6. Goudie S, McIntyre Z. A CRISIS WITHIN A CRISIS: The Impact of COVID-19 on Household Food Security Insights from Food Foundation. Surveys on how the Pandemic has Affected Food Access in the UK (March 2020 to January 2021). Food Foundation; 2021.

7. Group WB. Commodity Markets Outlook: The Impact of the War in Ukraine on Commodity Markets, April 2022. . 2022.

8. Statistics OfN. The cost of living, current and upcoming work: March 2022. Office for National Statistics; 2022.

9. Fuller E, Bankiewicz U, Davies B, Mandalia D, Stocker B. The Food and You Survey, Wave 5: Combined report for England, Wales and Northern Ireland. . In: Agency FS, editor. London2019.

10. Foundation. F. Millions of Parents Facing Food Insecurity Due to Soaring Energy Bills and Rising Food Prices. 2021.

11. Pettifer K, Patel M. Household Food Insecurity: Annex B to the FSA 22-06-09. . In: Agency FS, editor. Online2022.

12. Government announces Energy Price Guarantee for families and businesses while urgently taking action to reform broken energy market. [press release]. 8 September 2022 2022.

13. Watson M, Booth S, Velardo S, Coveney J. The Orthodox and Unorthodox Food Acquisition Practices and Coping Strategies Used by Food Insecure Adults: A Scoping Review. Journal of Hunger & Environmental Nutrition. 2022:1-16.

14. Leung CW, Epel ES, Ritchie LD, Crawford PB, Laraia BA. Food insecurity is inversely associated with diet quality of lower-income adults. J Acad Nutr Diet. 2014;114(12):1943-53.e2.

15. Nettle D, Andrews C, Bateson M. Food insecurity as a driver of obesity in humans: The insurance hypothesis. Behav Brain Sci. 2017;40:e105.

16. Seligman HK, Davis TC, Schillinger D, Wolf MS. Food insecurity is associated with hypoglycemia and poor diabetes self-management in a low-income sample with diabetes. J Health Care Poor Underserved. 2010;21(4):1227-33.

17. Andreae G, Scott S, Nguyen G, Bell Z, Mehmood H, Sermin-Reed L, et al. Food insecurity among pregnant women living in high-income countries: a systematic review. The Lancet. 2022;400:S17.

18. Laraia B, Epel E, Siega-Riz AM. Food insecurity with past experience of restrained eating is a recipe for increased gestational weight gain. Appetite. 2013;65:178-84.

19. Wiener RC, Sambamoorthi U, Shen C, Alwhaibi M, Findley P. Food Security and Unmet Dental Care Needs in Adults in the United States. J Dent Hyg. 2018;92(3):14-22.

20. Liu Y, Eicher-Miller HA. Food Insecurity and Cardiovascular Disease Risk. Curr Atheroscler Rep. 2021;23(6):24.

21. Nguyen BT, Shuval K, Bertmann F, Yaroch AL. The Supplemental Nutrition Assistance Program, Food Insecurity, Dietary Quality, and Obesity Among U.S. Adults. Am J Public Health. 2015;105(7):1453-9.

22. Fallaize R, Newlove J, White A, Lovegrove JA. Nutritional adequacy and content of food bank parcels in Oxfordshire, UK: a comparative analysis of independent and organisational provision. J Hum Nutr Diet. 2020;33(4):477-86.

23. Neter JE, Dijkstra SC, Visser M, Brouwer IA. Dutch food bank parcels do not meet nutritional guidelines for a healthy diet. Br J Nutr. 2016;116(3):526-33.

24. Hughes D, Prayogo E. A Nutritional Analysis of the Trussell Trust Emergency Food Parcel. The Trussell Trust; 2018.

25. Nagata JM, Gomberg S, Hagan MJ, Heyman MB, Wojcicki JM. Food insecurity is associated with maternal depression and child pervasive developmental symptoms in low-income Latino households. J Hunger Environ Nutr. 2019;14(4):526-39.

26. Martin MS, Maddocks E, Chen Y, Gilman SE, Colman I. Food insecurity and mental illness: disproportionate impacts in the context of perceived stress and social isolation. Public Health. 2016;132:86-91.

27. Ding M, Keiley MK, Garza KB, Duffy PA, Zizza CA. Food insecurity is associated with poor sleep outcomes among US adults. J Nutr. 2015;145(3):615-21.

28. Wang Q. Food Insecurity and Sleep Disturbance Among 223,561 Adolescents: A Multi-Country Analysis of Cross-Sectional Surveys. Front Public Health. 2021;9:693544.

29. Smith J, Stevens H, Lake AA, Teasdale S, Giles EL. Food insecurity in adults with severe mental illness: A systematic review with meta-analysis. Journal of Psychiatric and Mental Health Nursing. 2023;00(n/a):1-19.

30. Men F, Elgar FJ, Tarasuk V. Food insecurity is associated with mental health problems among Canadian youth. J Epidemiol Community Health. 2021;75(8):741-8.

31. Pollard CM, Booth S. Food Insecurity and Hunger in Rich Countries-It Is Time for Action against Inequality. Int J Environ Res Public Health. 2019;16(10).

32. Trust T. End of Year Stats. Online: Trussell Trust; 2022 [Available from: <https://www.trusselltrust.org/news-and-blog/latest-stats/end-of-year-stats-ob/>.

33. DWP. Family Resources Survey: financial year 2020 to 2021: Results from the Family Resources Survey for the financial year 2020 to 2021, providing information on income and circumstances of UK households. In: Pensions DoWa, editor. Online: UK Government; 2021.

34. Johnson CM, Sharkey JR, Lackey MJ, Adair LS, Aiello AE, Bowen SK, et al. Relationship of food insecurity to women's dietary outcomes: a systematic review. Nutr Rev. 2018;76(12):910-28.

35. Dolin CD, Compher CC, Oh JK, Durnwald CP. Pregnant and hungry: addressing food insecurity in pregnant women during the COVID-19 pandemic in the United States. Am J Obstet Gynecol MFM. 2021;3(4):100378.

36. Cukrowska-Torzewska E, Matysiak A. The motherhood wage penalty: A meta-analysis. Social Science Research. 2020;88-89:102416.

37. Ivers LC, Cullen KA. Food insecurity: special considerations for women. Am J Clin Nutr. 2011;94(6):1740s-4s.

38. McIntyre L, Glanville NT, Raine KD, Dayle JB, Anderson B, Battaglia N. Do low-income lone mothers compromise their nutrition to feed their children? Cmaj. 2003;168(6):686-91.

39. Shinwell J, Defeyter MA. Food Insecurity: A Constant Factor in the Lives of Low-Income Families in Scotland and England. Front Public Health. 2021;9:588254.

40. Armstrong B, Hepworth AD, Black MM. Hunger in the household: Food insecurity and associations with maternal eating and toddler feeding. Pediatr Obes. 2020;15(10):e12637.

41. Ward WL, Swindle TM, Kyzer AL, Edge N, Sumrall J, Whiteside-Mansell L. Maternal Depression: Relationship to Food Insecurity and Preschooler Fruit/Vegetable Consumption. Int J Environ Res Public Health. 2019;17(1).

42. Reesor-Oyer L, Cepni AB, Lee CY, Zhao X, Hernandez DC. Disentangling food insecurity and maternal depression: which comes first? Public Health Nutr. 2021;24(16):5506-13.

43. Kominiarek MA, Rajan P. Nutrition Recommendations in Pregnancy and Lactation. Med Clin North Am. 2016;100(6):1199-215.

44. Marangoni F, Cetin I, Verduci E, Canzone G, Giovannini M, Scollo P, et al. Maternal Diet and Nutrient Requirements in Pregnancy and Breastfeeding. An Italian Consensus Document. Nutrients. 2016;8(10).

45. Maynard M, Andrade L, Packull-McCormick S, Perlman CM, Leos-Toro C, Kirkpatrick SI. Food Insecurity and Mental Health among Females in High-Income Countries. Int J Environ Res Public Health. 2018;15(7).

46. Gluckman PD, Hanson MA, Cooper C, Thornburg KL. Effect of in utero and early-life conditions on adult health and disease. N Engl J Med. 2008;359(1):61-73.

47. Wadhwa PD, Buss C, Entringer S, Swanson JM. Developmental origins of health and disease: brief history of the approach and current focus on epigenetic mechanisms. Semin Reprod Med. 2009;27(5):358-68.

48. Arima Y, Fukuoka H. Developmental origins of health and disease theory in cardiology. J Cardiol. 2020;76(1):14-7.

49. Wasti SP, Simkhada P, van Teijlingen ER, Sathian B, Banerjee I. The Growing Importance of Mixed-Methods Research in Health. Nepal J Epidemiol. 2022;12(1):1175-8.

50. Shorten A, Smith J. Mixed methods research: expanding the evidence base. Evidence Based Nursing. 2017;20(3):74-5.

51. Bickel G, Nord M, Price C, Hamilton W, Cook J. Guide to Measuring Household Food Security, Revised 2000. In: In United States Department of Agriculture FaNS, editor. 2000.

52. Calloway EE, Carpenter LR, Gargano T, Sharp JL, Yaroch AL. New measures to assess the “Other” three pillars of food security–availability, utilization, and stability. International Journal of Behavioral Nutrition and Physical Activity. 2023;20(1):51.

53. Beacom E, Furey S, Hollywood L, Humphreys P. Investigating food insecurity measurement globally to inform practice locally: a rapid evidence review. Crit Rev Food Sci Nutr. 2021;61(20):3319-39.

54. Nguyen G. Measurement of household food insecurity in the United Kingdom [Doctoral thesis]: University of Aberdeen; 2020.

55. Roberts K, Dawson J, Cade J, Holdsworth M. Using dietary patterns methods to identify indicators of diet quality in the UK adult population – the development and validation of Brief Diet Quality Assessment Tools (BDQAT)2019; 1(RFC1.2).

56. Wisner KL, Parry BL, Piontek CM. Clinical practice. Postpartum depression. N Engl J Med. 2002;347(3):194-9.

57. Cox JL, Holden JM, Sagovsky R. Detection of postnatal depression. Development of the 10-item Edinburgh Postnatal Depression Scale. Br J Psychiatry. 1987;150:782-6.

58. Dewa LH, Lawrence-Jones A, Crandell C, Jaques J, Pickles K, Lavelle M, et al. Reflections, impact and recommendations of a co-produced qualitative study with young people who have experience of mental health difficulties. Health Expect. 2021;24 Suppl 1(Suppl 1):134-46.

59. Smithson H, Jones A. Co-creating youth justice practice with young people: Tackling power dynamics and enabling transformative action. Children & Society. 2021;35(3):348-62.

60. Smith B, Williams O, Bone L, Collective tMSWC-p. Co-production: A resource to guide co-producing research in the sport, exercise, and health sciences. Qualitative Research in Sport, Exercise and Health. 2023;15(2):159-87.

61. Lundy L. ‘Voice’ is not enough: conceptualising Article 12 of the United Nations Convention on the Rights of the Child. British Educational Research Journal. 2007;33(6):927-42.

62. O’Cathain A, Murphy E, Nicholl J. Three techniques for integrating data in mixed methods studies. BMJ. 2010;341:c4587.

63. Farmer T, Robinson K, Elliott SJ, Eyles J. Developing and implementing a triangulation protocol for qualitative health research. Qual Health Res. 2006;16(3):377-94.

64. Kunst N, Wilson ECF, Glynn D, Alarid-Escudero F, Baio G, Brennan A, et al. Computing the Expected Value of Sample Information Efficiently: Practical Guidance and Recommendations for Four Model-Based Methods. Value in Health. 2020;23(6):734-42.

65. Heath A, Manolopoulou I, Baio G. Efficient Monte Carlo Estimation of the Expected Value of Sample Information Using Moment Matching. Med Decis Making. 2018;38(2):163-73.

66. Heath A. Calculating Expected Value of Sample Information Adjusting for Imperfect Implementation. Med Decis Making. 2022;42(5):626-36.

67. Heath A, Manolopoulou I, Baio G. Estimating the Expected Value of Sample Information across Different Sample Sizes Using Moment Matching and Nonlinear Regression. Med Decis Making. 2019;39(4):346-58.

68. Braun V, Clarke V. To saturate or not to saturate? Questioning data saturation as a useful concept for thematic analysis and sample-size rationales. Qualitative Research in Sport, Exercise and Health. 2021;13(2):201-16.

# Appendices

## Appendix 1- Required documentation

1. PIs signed CV and a copy of their GCP certificate
2. Participant Information Sheets and consent forms on letter headed paper
3. Confirmation of capacity and capability or equivalent
4. Delegation log

## Appendix 2 – Schedule of Procedures

| **Procedures** | **Visits** | | |
| --- | --- | --- | --- |
|  | **Screening medical records** | **3rd trimester** | **3^rd^ trimester or up to 12 months postnatal** |
| Recruitment packs prepared and distributed to eligible patients | x |  |  |
| Paper or online questionnaire completion |  | x |  |
| University research team identify food insecure people to invite for interview |  |  | x |
| Informed consent |  |  | x |
| Interviews |  |  | x |

## Appendix 3 – Amendment History

List details of all protocol amendments here whenever a new version of the protocol is produced.

Protocol amendments must be submitted to the Sponsor for approval prior to submission to the REC.

| **Amendment No.** | **Protocol version no.** | **Date issued** | **Author(s) of changes** | **Details of changes made** |
| --- | --- | --- | --- | --- |
| 1 | 2.0 | 18-March-24 | Kiya Hurley | The following additions were made following ethical review:   - Description of the sample size calculation - Definition of end of study - Statement that the audio-recordings will be deleted. |
| 2 | 3.0 | 16-Apr-24 | Kiya Hurley | The following changes were made:   - Updated information on the process for dealing with missing information - Addition of Dr Charlotte Wilson to the Advisory Board and Dr Zainab Akhter and Dr Ella Dyer to the research team - Minor typographical errors corrected |
